# Establishment of serine protease *htrA* mutants in *Helicobacter pylori* is associated with *secA* mutations

**DOI:** 10.1038/s41598-019-48030-6

**Published:** 2019-08-13

**Authors:** Anna Zawilak-Pawlik, Urszula Zarzecka, Dorota Żyła-Uklejewicz, Jakub Lach, Dominik Strapagiel, Nicole Tegtmeyer, Manja Böhm, Steffen Backert, Joanna Skorko-Glonek

**Affiliations:** 1 Hirszfeld Institute of Immunology and Experimental Therapy, Polish Academy of Sciences, Department of Microbiology, Weigla 12, 53–114 Wrocław, Poland; 2 Department of General and Medical Biochemistry, Faculty of Biology, University of Gdańsk, Wita Stwosza 59, 80–308 Gdańsk, Poland; 3 Department of Biology, Division of Microbiology, Friedrich-Alexander University Erlangen-Nuremberg, Staudtstr. 5, 91058 Erlangen, Germany; 4 BBMRI.pl Consortium, Stabłowicka 147, 54–066 Wrocław, Poland; 5 Biobank Lab, Department of Molecular Biophysics, Faculty of Biology and Environmental Protection, University of Lodz, Pilarskiego 14, Lodz, Poland

**Keywords:** Functional genomics, Cellular microbiology, Post-translational modifications

## Abstract

*Helicobacter pylori* plays an essential role in the pathogenesis of gastritis, peptic ulcer disease, and gastric cancer. The serine protease HtrA, an important secreted virulence factor, disrupts the gastric epithelium, which enables *H*. *pylori* to transmigrate across the epithelium and inject the oncogenic CagA protein into host cells. The function of periplasmic HtrA for the *H*. *pylori* cell is unknown, mainly due to unavailability of the *htrA* mutants. In fact, *htrA* has been described as an essential gene in this bacterium. We have screened 100 worldwide *H*. *pylori* isolates and show that only in the N6 strain it was possible to delete *htrA* or mutate the *htrA* gene to produce proteolytically inactive HtrA. We have sequenced the wild-type and mutant chromosomes and we found that inactivation of *htrA* is associated with mutations in SecA – a component of the Sec translocon apparatus used to translocate proteins from the cytoplasm into the periplasm. The cooperation of SecA and HtrA has been already suggested in *Streptococcus pneumonia*, in which these two proteins co-localize. Hence, our results pinpointing a potential functional relationship between HtrA and the Sec translocon in *H*. *pylori* possibly indicate for the more general mechanism responsible to maintain bacterial periplasmic homeostasis.

## Introduction

HtrA (High Temperature Requirement A) proteins are a family of evolutionarily well preserved serine proteases, identified in the majority of the examined organisms. In addition to their proteolytic activity, several HtrAs exhibit also chaperone-like properties. In prokaryotes these proteins localize to the periplasm, may be attached to membranes or are secreted out of the cell^1^. Most of HtrAs are involved in the protein quality control and are responsible for the removal of improperly folded proteins from the cellular envelope using their both, proteolytic and chaperone-like, activities^2^. Aside from their housekeeping functions, certain HtrA homologs play regulatory roles, e.g., regulate the σ^E^-dependent stress response^3^, others are involved in maturation and secretion of surface proteins, including several virulence factors^4^. In the Gram-positive bacteria, *Streptococcus pyogenes* and *Streptococcus pneumoniae*, HtrA co-localizes to the Sec machinery on the cell surface^5–7^. This fact raises the possibility that HtrA is involved in processing/maturation and/or in quality control of the exported proteins in streptococci. Recently, it was demonstrated that secreted fractions of bacterial HtrAs can directly contribute to the pathogenesis of certain human diseases^8^. Such diverse tasks are usually performed by several HtrA homologs that function in parallel in a cell. For example, bacteria from the Enterobacteriaceae family encode three members of the HtrA family: HtrA (DegP), DegQ (HhoA) and DegS (HhoB) (UniprotKB, www.expasy.org). However, there are various other bacterial species that carry only one HtrA homolog, including the human pathogen *Helicobacter pylori*.

*H*. *pylori* secretes a certain fraction of HtrA into the extracellular space as an active soluble protease or embedded in outer membrane vesicles (OMVs), promoting bacterial colonization and invasion of host tissues^9–12^. Remarkably, secreted HtrA can cleave the extracellular NTF (N-terminal fragment) domain of E-cadherin, the adherens junction protein in polarized gastric epithelial cells^11,13^. In fact, the ectodomain cleavage of E-cadherin by HtrA results in the disruption of the intercellular adherens junction complex, which supports *H*. *pylori* access to deeper tissues^11,14^. In addition, *H*. *pylori* HtrA also targets the tight junction proteins occludin and claudin-8, and the extracellular matrix protein fibronectin^11,14,15^. Moreover, we demonstrated that HtrA protease activity is required for paracellular transmigration of *H*. *pylori* to access basolateral sites, target integrin-α_5_β_1_ and inject oncogenic protein CagA via a type IV secretion system encoded by the *cag* pathogenicity island (PAI)^11,14,15^. Thus, secreted HtrA exemplifies the first known non-PAI protein contributing considerably to T4SS functionality in *H*. *pylori*.

Significant research progress on *H*. *pylori* HtrA properties is mainly hindered by the non-existence of *htrA* knockout mutants, advocating that *htrA* may be an essential gene in *H*. *pylori*^16^. To by-pass this major draw-back and to investigate the role of HtrA in more detail, in previous studies we overexpressed the enzyme by introducing a second *htrA* gene copy in the genome and investigated multiple virulence activities by *H*. *pylori*^17^, and established a genetic complementation system of *H*. *pylori* HtrA in the close relative *Campylobacter jejuni* showing its functionality in the bacteria^18^. Still, an appropriate model to study the HtrA function in *H*. *pylori* was missing.

In the present work, we demonstrated that in one strain, *H*. *pylori* N6, it was possible to delete the *htrA* gene from the chromosome or to introduce a 661T > G point mutation into *htrA* which changes serine 221 into alanine (S221A) and, in consequence, destroys the catalytically active site in HtrA. We also showed that *H*. *pylori* lacking HtrA or with inactive HtrA exhibited reduced transmigration activity across MKN28 polarized epithelial cells and reduced translocation of CagA in polarised Caco-2 cells in comparison to the wild-type or *htrA*-complemented strains. Whole genome sequencing demonstrated that in both mutant strains, the *htrA* deletion knockout (N6 Δ*htrA*) or the protease deficient (N6 *htrA*S221A), *htrA* inactivation was accompanied by the spontaneous occurrence of an additional mutation in the *secA* gene. The function of the SecA protein in *H*. *pylori* was not studied thoroughly, but its homologs from other bacterial species are engaged in the transport of newly synthesized polypeptides to be exported by the Sec translocon (type II secretion system). Thus, our work led to construction of a highly valuable new tool for further studies on the role of HtrA in *H*. *pylori* physiology and virulence, but also indicated for the possible interdependence between Sec translocon and HtrA in *H*. *pylori* in maintaining proper homeostasis in the *H*. *pylori* periplasm.

## Results

### *htrA* can be deleted in the *H. pylori* N6 strain

**Figure 1:**
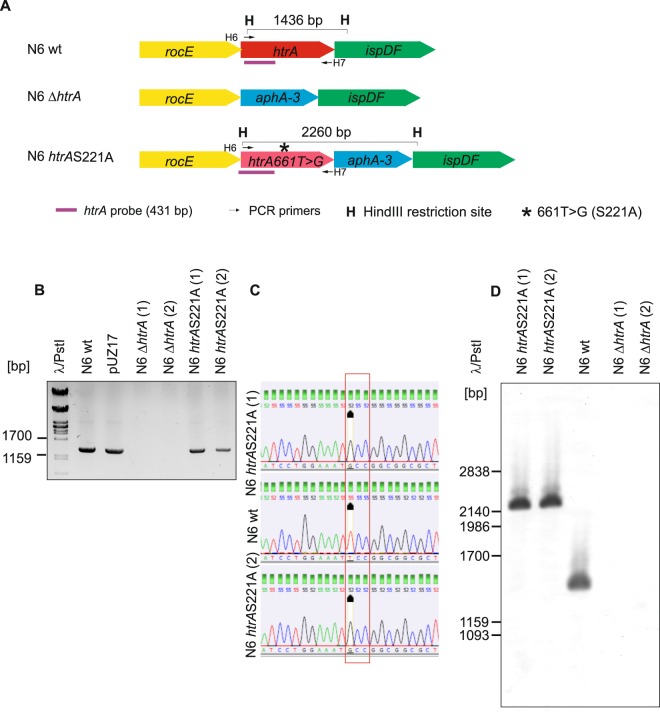
Analysis of *H*. *pylori* Δ*htrA* and *htrA*S221A mutated strains. (1) and (2) denotes two independent clones. (**A**) Schematic presentation of a chromosomal region *rocE*-*ispDF* in *H*. *pylori* wild-type and *htrA*-mutated strains. The genes are not drawn to scale. Features most important for the analysis of the mutated strains are depicted. (**B**) PCR analysis of genomic DNA isolated from *H*. *pylori* wild-type and mutant strains. H6-H7 primer pair (S2 Table) was used to amplify *htrA* (1428 bp) in constructed mutants. (**C**) Analysis of 661T > G *htrA* mutation in *H*. *pylori htrA*S221A mutant strain. *htrA* was amplified by H8-H9 primer pair and sequenced by Sanger sequencing. TCC/GCC (S221/A221) codons are boxed and 661T > G mutation is highlighted. (**D**) Southern blot analysis of Δ*htrA* and *htrA*S221 A strains. Genomic DNA, digested with HindIII restriction enzyme, were resolved in 1% agarose gel, transferred onto a nylon membrane and probed with the digoxigenin-labeled DNA probe (**A**). Southern blot was developed by anti-digoxigenin antibody followed by colorimetric reaction. The full-length gels/blots are presented in Supplementary Fig. S8.

HtrA has been shown to be essential for the viability of every *H*. *pylori* strain tested so far^16,19^. However, it is not very common that HtrA is indispensable for many other bacteria under conventional growth conditions. Thus, we undertook another attempt to inactivate *htrA* in *H*. *pylori* using two strategies. We constructed two vectors, pUZ16 and pUZ17, which were designed to recombine with the *H*. *pylori* chromosome at the *htrA* locus and subsequently delete *htrA* or substitute T661 for G, respectively (Methods and Supplementary Fig. S1). 661T > G introduces the S221A mutation and, in consequence, inactivates the HtrA catalytic active site. A set of 100 worldwide *H*. *pylori* strains from Europe, Asia, Australia, North America and South America (Supplementary Table S1) was transformed with purified pUZ16 and pUZ17 plasmids. Kanamycin resistant colonies were only obtained for the N6 strain. Genomic DNA was extracted from eight pUZ16 and ten pUZ17 transformants and analyzed by PCR using H6-H7 primers (Supplementary Table S4) which amplify the entire *htrA* gene. The analysis indicated that no *htrA* gene was present anymore in six *H*. *pylori htrA* deletion (N6 Δ*htrA*) clones, while *htrA* was detected in the *H*. *pylori* strain with S221A mutation (N6 *htrA*S221A) (Fig. 1AB and Fig. S2). The PCR products, amplified using H8-H9 primer pair and *H*. *pylori* N6 *htrA*S221A genomic DNA as a template, were PdiI digested and/or sequenced, which confirmed that in eight clones the *htrA* locus contained the 661T > G substitution introduced into *htrA* (Fig. 1C and Fig. S2). Finally, using two independent clones of each mutant type, we performed Southern blotting, which confirmed that recombination occurred at the desired locus in both N6 Δ*htrA* and N6 *htrA*S221A strains and that no additional copy of *htrA* was present in the *H*. *pylori* wild-type or mutant strains (Fig. 1D).

**Figure 2:**
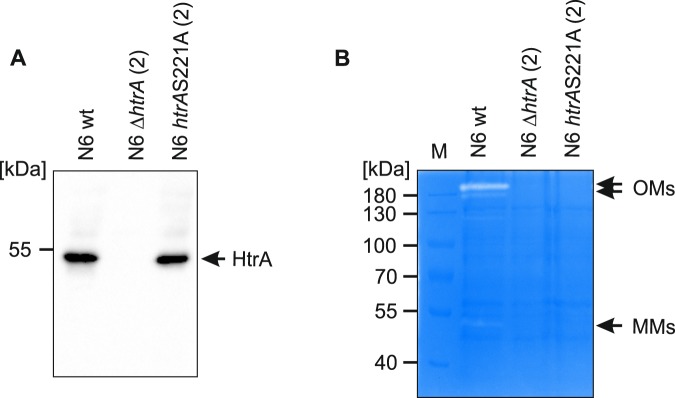
Analysis of HtrA synthesis and activity in *H*. *pylori* N6 wild-type and mutant strains. (**A**) Western blot analysis of HtrA in *H*. *pylori* strains. A rabbit polyclonal anti-HtrA IgG was used to detect HtrA (51 kDa) in bacterial lysates. (**B**) The ability to cleavage of casein was analyzed by zymography. The position of proteolytically active HtrA monomers (MMs) and oligomers (OMs) is indicated. The full-length gels/blots are presented in Supplementary Fig. S8.

Next, we performed western blotting and casein zymography analyses to confirm the presence or absence of HtrA in our *H*. *pylori* clones (Fig. 2). No HtrA protein was detected in the N6 Δ*htrA* strain by the anti-HtrA antibodies. The corresponding protein lysate of this mutant did not show any proteolytic activity against casein. We also confirmed that the HtrAS221A variant, which was detected by western blotting in the *H*. *pylori htrA*S221A strain, showed no caseinolytic activity. Casein zymography also revealed that no detectable additional caseinolytic protease was present in the *H*. *pylori* N6 Δ*htrA* strain. Thus, to summarize, all performed analyses confirmed that *htrA* was successfully deleted in *H*. *pylori* N6 Δ*htrA*, while it was correctly exchanged for the mutated gene in *H*. *pylori* N6 *htrA*S221A.

### Deletion or mutation of the *htrA* gene selects for suppressor mutations in the *secA* gene

N6 represents the only *H*. *pylori* strain, in which *htrA* was successfully inactivated thus far. These surprising findings raised questions about the uniqueness of this isolate, but also about possible suppressor mutations that might have been selected by inactivation of *htrA*. Since the full *H*. *pylori* N6 sequence is not available in the format allowing for extensive *in silico* analyses, and taking into account that our laboratory stock of N6 might have accumulated some spontaneous mutations upon passaging, we decided to sequence the entire genomes of the N6 Δ*htrA* (clone 2) and N6 *htrA*S221A (clone 2) mutant strains and the corresponding parental wild-type strain.

Using OrthoVenn^20^, we compared the N6 wild-type encoded proteome with proteomes of 5 other fully sequenced *H*. *pylori* strains: 26695, J99, India7, Shi470, and HPAG1, which represent strains in which no *htrA* mutant was obtained. The analysis revealed that the N6 wild-type strain contained genes that were found in at least one of the 5 sequenced genomes (Fig. S3). Thus, there were no additional proteins (e.g., proteases) in the N6 wild-type strain that could have taken over the function of HtrA allowing for *htrA* deletion.

**Figure 3:**
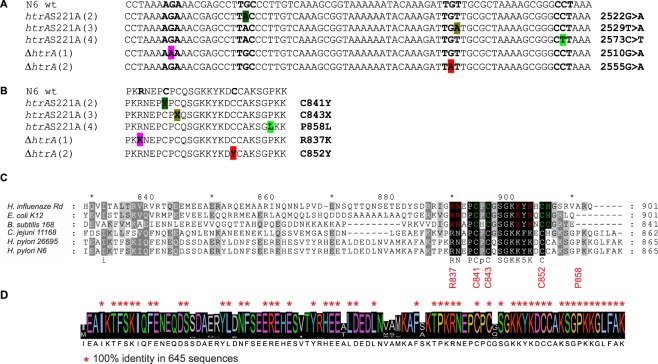
Comparison of *secA* sequences. (**A**) 2503–2568 nt of *H*. *pylori secA* nucleotide sequences in wild-type and mutant strains. Detected nucleotide substitutions are highlighted; the codon, in which the substitution occurred, is presented in bold. (**B**) Amino acid sequence of *H*. *pylori* SecA (835–856 aa) in wild-type and mutant strains. Residues in which substitution occurred are in bold, while substitutions are highlighted. (**C**) Sequence alignment of the C-terminal regions of SecA proteins from selected bacterial species. Residues that contact SecB are red, whereas those involved in zinc-binding coordination are green^20^. Residues mutated in *H*. *pylori* SecA are marked below the alignment. (**D**) The amino acid sequence conservation *H*. *pylori* SecA sequences. 648 *H*. *pylori* SecA sequences were retrieved by Protein BLAST search from Genbank; 3 sequences were discarded because they were too short to be aligned. Residues mutated in *H*. *pylori* SecA are marked above the alignment.

Next, we compared sequences of *H*. *pylori* N6 wild-type and mutant strains. We identified genetic variants in the *H*. *pylori* N6 *htrA*S221A and N6 Δ*htrA* strains based on short reads mapping result. Surprisingly, in both strains we detected single missense mutations in the *secA* gene, 2522G > A or 2555G > A, which resulted in the C841Y or C852Y substitutions in the SecA protein, respectively. No other mutations were identified in any of the strains. To further confirm the presence of mutations, *secA* of *H*. *pylori* wild-type, N6 *htrA*S221A and N6 Δ*htrA* were amplified by PCR using H10-H11 primer pair and sequenced by the Sanger method. Sequencing confirmed the presence of distinct mutations detected by NGS sequencing (Fig. 3). Additional Sanger sequencing of another clone of *H*. *pylori* N6 Δ*htrA* (clone 1) and two other clones of N6 *htrA*S221A (clones 3 and 4) revealed other types of mutation, 2510G > A, 2529T > A and 2573C > T resulting in the SecA R837K, C843X and P858L mutant protein variants (Fig. 3). All five mutations are located within the C-terminal part of SecA, which in other bacteria is responsible for interaction with the SecB chaperone and Zn^2+^ binding^21^ (see Discussion). Finally, the *secA* gene sequences of the mutant strains were compared with worldwide *H*. *pylori* strains deposited in the RefSeq NCBI database. Remarkably, in a total of 645 *H*. *pylori* SecA sequences analyzed, similar mutations at the above positions do not occur and are unique to the N6 Δ*htrA* and N6 *htrA*S221A clones (Fig. 3D). Hence, the presence of these mutations must be directly associated with a lack of the functional HtrA protease.

**Figure 4:**
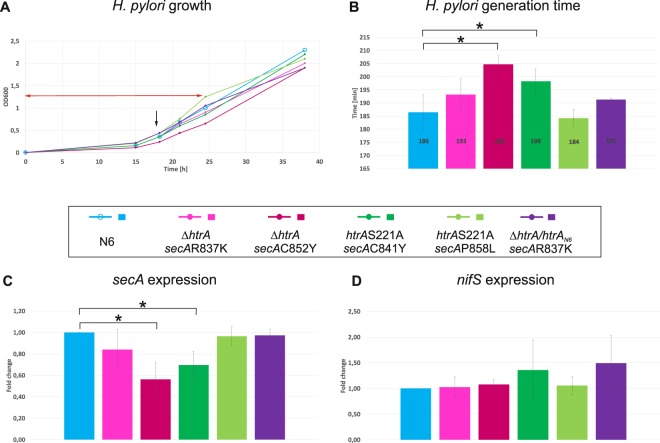
*H*. *pylori* growth and *secA* expression in *htrA*/*secA* double mutant strains. (**A**) Growth curve analysis of *H*. *pylori* N6 wild-type and mutant strains. Two different *secA* mutant strains of each *htrA* mutant type were analysed (see Fig. 3 and Table S2 for *secA* mutant characteristics). *H*. *pylori* was inoculated in Brucella broth to OD_600_ = 0.005 and cultured until a stationary phase of growth. The red arrow indicates time period used in generation time calculations (**B**), while the black arrow indicates a time-point of sample collection for RNA isolation and *secA* expression analysis (**C**). All four independent analyses of *H*. *pylori* growth are shown in Fig. S5. (**B**) Generation times were calculated for *H*. *pylori* wild type, Δ*htrA*, *htrA*S221A and Δ*htrA/htrA*_N6_ mutant strains grown in four independent cultures for time periods indicated by the red arrows (A and Fig. S5); three independent cultures were grown for Δ*htrA*/*htrA*_N6_. (**C**,**D**) RT-qPCR analysis of *secA* and *nifS* expression in *H*. *pylori* N6 wild-type and mutant strains. Results show fold changes (mean +/−  standard deviation) of *secA* or *nifS* expression relative to N6 wild-type strain. 16SrRNA was used as a reference gene. Three biological replicates were used for the analysis. Statistical significance was calculated using the t-test *p < 0.05.

To better characterize the *H*. *pylori* double mutant strains, we analyzed growth of two independent clones of each *htrA* mutant type (Δ*htrA* and *htrA*S221A), each containing a different *secA* mutation, and compared it to the growth of the wild-type strain and the complementation mutant Δ*htrA/htrA*_*N6*_ that was derived from ∆*htrAsecA*R837K (clone 1), (for strain construction details see Methods, Supplementary Fig. S4 and Table S2). The analysis of growth curves and average generation time calculated for the first 24–27 hours of growth (i.e., characterizing both the lag phase and logarithmic phase of growth) showed that *H*. *pylori* ∆*htrAsecA*R837K and *htrA*S221A*secA*P858Y grew similarly to the wild-type and Δ*htrA/htrA*_*N6*_ strains, while the ∆*htrAsecA*C852Y *htrA*S221A*secA*C841Y grew significantly slower (Fig. 4AB and Fig. S5). Since the two clones of each Δ*htrA* or *htrA*S221A mutant type had the same *htrA* mutation but differed in *secA* mutation, the differences in growth rate were possibly caused by the type of the *secA* mutation. R837K and P858L mutations are possibly less harmful to *H*. *pylori* than C852Y and C841Y mutations. We also analyzed *secA* expression in each of the mutated strain. The *secA* expression level was lower in *H*. *pylori* mutants with retarded growth, while in mutants with undisturbed growth *secA* expression was similar to the wild-type and Δ*htrA/htrA*_*N6*_ strains. To verify whether lower *secA* expression was not biased due to delayed growth, we analyzed expression of *nifS*, a gene encoding cysteine desulfurase - an enzyme unrelated to secretion system. The expression of *nifS* was similar in the wild-type and all mutant strains. Thus, we conclude that inactivation of *htrA* by deletion or mutation of its catalytic center is associated with two types of suppressor mutation in *secA*: 1/ affecting *H*. *pylori* growth and *secA* expression, and 2/ not affecting *H*. *pylori* growth and *secA* expression (both tested under optimal growth conditions). We did not find any mutation in the *secA* promoter regions in the two sequenced mutants Δ*htrAsecA*C852 and *htrA*S221A*secA*C841Y, each of which is characterized by retarded growth and reduced *secA* expression that could affect *secA* expression. However, we did not sequence chromosomes of strains, in which growth and *secA* expression were similar to the wild-type strain, thus we can’t exclude a second type or suppressor mutation. The molecular mechanism of growth retardation and *secA* expression reduction remains to be discovered. Nonetheless, our comprehensive analyses allowed to select ∆*htrAsecA*R837K or *htrA*S221AsecAP858L strains, which can be used for further HtrA studies without secondary effects of *secA* mutation. In our further phenotypic studies, we used derivatives of *H*. *pylori* N6 ∆*htrAsecA*R837K.

### Inactivation of HtrA decreases bacterial transmigration across polarized epithelial cells

**Figure 5:**
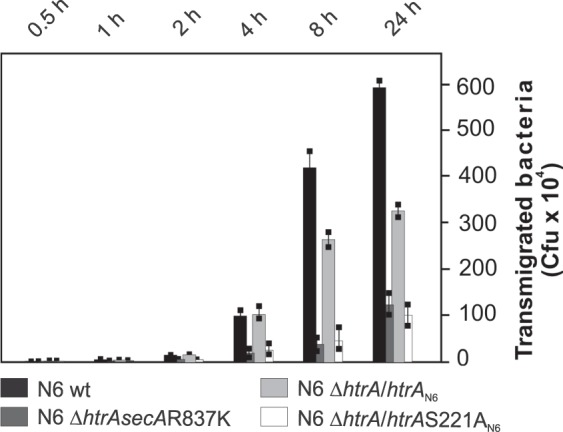
Downregulation of bacterial transmigration by *H*. *pylori htrA* mutations. Polarized monolayers of MKN28 cells were infected by *H*. *pylori* wild-type or mutant strains. The mutant strains (N6 Δ*htrA*, N6 Δ*htrA*/*htrA*_N6_ and N6 Δ*htrA*/*htrA*S221A_N6_ encode the same SecA R837K mutant variant). Transmigrated bacteria were quantified by counting CFUs. All experiments were done in triplicates.

To assess the usefulness of the constructed *H*. *pylori htrA* mutant strains as novel tools to study the role of HtrA in the pathogenesis of *H*. *pylori* infections, we determined the transmigration rates by the *H*. *pylori* wild-type and mutated strains (Fig. 5). For this purpose, the polarized MKN28 cells were grown in a transwell filter system for 14 days to reach confluent monolayers. The cells were infected with *H*. *pylori* for 24 h in the apical chamber. Transmigrated bacteria were harvested from the bottom chamber, grown on GC agar plates, and the CFUs were determined (Fig. 5). The results show that after 24 hrs the number of transmigrated wild-type *H*. *pylori* bacteria were approx. 600 × 10^4^. The number of transmigrated *H*. *pylori* N6 Δ*htrAsecA*R837K bacteria were about 100 × 10^4^ CFU and decreased by approximately 6-fold compared to the corresponding control *H*. *pylori* wild-type strain. As further controls, we analysed transmigration of *H*. *pylori htrA* complemented strain (N6 Δ*htrA*/*htrA*_N6_) or a strain in which the *htrA* 661T > G mutated gene was introduced into *htrA* wild-type chromosomal locus (N6 Δ*htrA*/*htrA*S221A_N6_) (both strains encode SecAR837K variant, see Methods, Supplementary Fig. S4, Fig. S6 and Table S2). The number of transmigrated *H*. *pylori* N6 Δ*htrA*/*htrA*_N6_ bacteria were approx. 300 × 10^4^ (Fig. 5). This indicated that wild-type *htrA* reintroduced into N6 Δ*htrA* strain restored *H*. *pylori* transmigration ability to 50% of the corresponding wild-type strain. Transmigration of N6 Δ*htrA*/*htrA*S221A_N6_ bacteria was similar to transmigration of N6 Δ*htrAsecA*R837K, thus HtrAS221A protein variant cannot complement the *htrA* deletion in this process. The fully active complementation of N6 Δ*htrA* was probably not possible due to the presence of a suppressor mutation in *secA* gene. Nonetheless, we could show that proteolytically active *HtrA* is important for *H*. *pylori* transmigration and that the constructed strains can be used as valuable tools in further studies on *H*. *pylori* HtrA.

### Inactivation of HtrA does not affect bacterial host cell binding, but inhibits translocation and phosphorylation of CagA in polarized Caco-2 cells vs. non-polarized AGS cells

**Figure 6:**
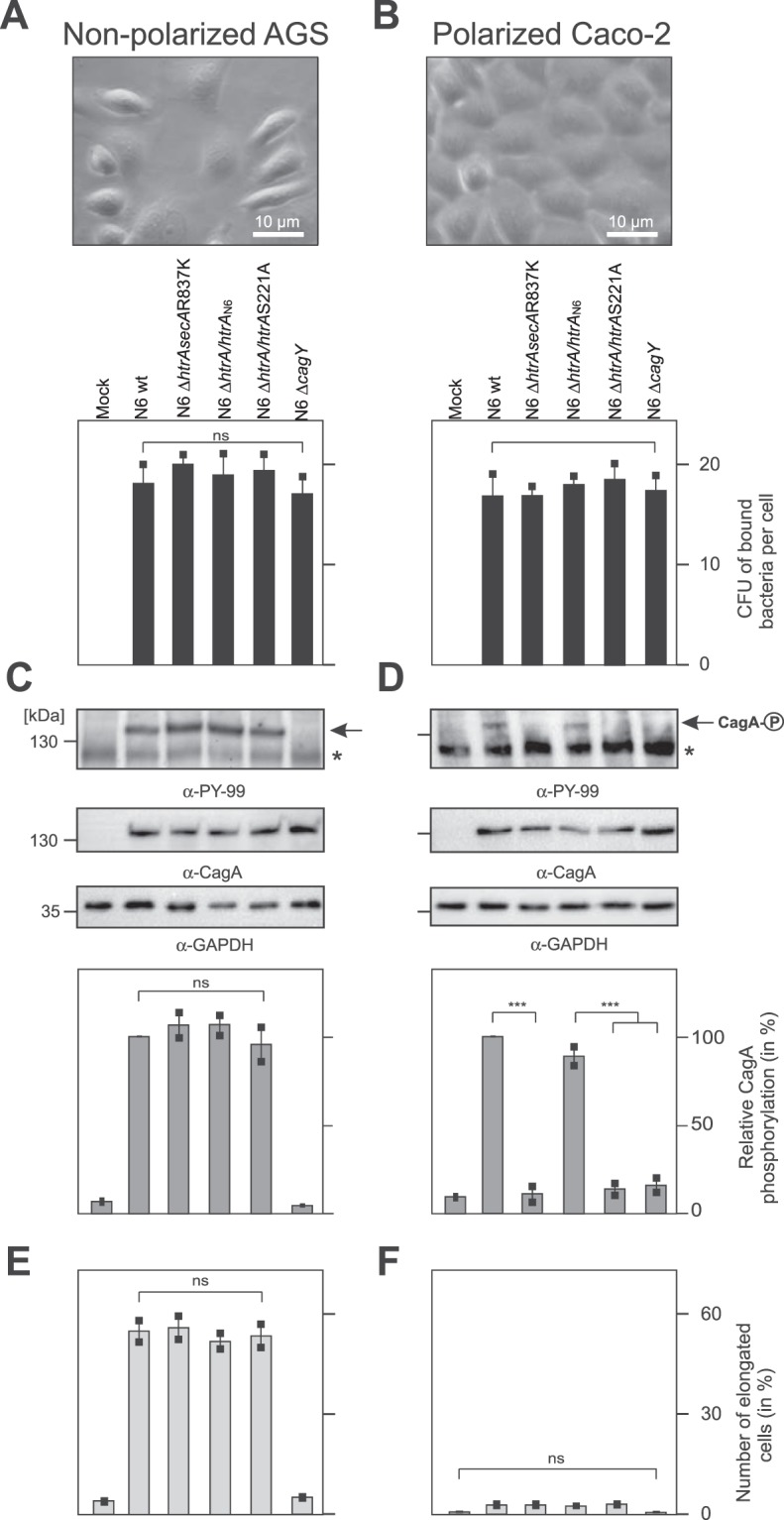
Analysis of *H*. *pylori* wild-type and *htrA* mutated strains translocation and phosphorylation of CagA in non-polarized AGS cells and in polarized Caco-2 cells. (**A**,**B**) Phase contrast microscopy and quantification of bacterial cell binding of non-polarized AGS cells (**A**) and polarized monolayers of Caco-2 cells (**B**). (**C**,**D**) Translocation and phosphorylation of CagA were monitored by western blotting after 6 hours of infection using an MOI of 25. Anti-PY-99 antibody recognises proteins with phosphorylated tyrosine residues, including CagA, that has been translocated into the host cell and phosphorylated at EPIYA motif. The asterisks mark a 130 kDa host cell protein running below phospho-CagA. Phospho-CagA was marked with arrows. Anti-CagA antibody recognises CagA regardless of its phosphorylation status. The relative phosphorylation of CagA was quantified densitometrically. (**E**,**F**) Quantification of the elongation (hummingbird) phenotype in infected cells. All mutated *htrA* strains are described in the text. The Δ*cagY* mutant (*cagY* is an ortholog of *virB10*) has a T4SS defect and served as negative control. All experiments were done in triplicates. The full-length blots are presented in Supplementary Fig. S10.

Next, we investigated if inactivation of the *htrA* gene may affect bacterial binding to host cells and translocation of CagA. For this purpose, non-polarized AGS cells in comparison to polarized Caco-2 cells were infected by the above described *H*. *pylori* strains for 6 hours using an MOI of 25 (Fig. 6). The results show that bacterial cell binding was very effective by *H*. *pylori* wild-type and was not inhibited by mutation of *htrA* (Fig. 6AB). Remarkably, translocation and phosphorylation of CagA were very high and similar between the strains in infected AGS cells (Fig. 6C), but deletion of *htrA* or infection with the protease-inactive Δ*htrA*/*htrA*S221A_N6_ variant abolished this effect in polarized Caco-2 cells (Fig. 6D). Successful translocation and phosphorylation of CagA was accompanied by the induction of the elongation (hummingbird) phenotype in infected AGS cells (Fig. 6E), while this phenotype was not expressed in polarized Caco-2 cells, irrespectively of whether CagA was phosphorylated or not (Fig. 6F).

## Discussion

In the majority of bacterial species, mutations in the *htrA* gene are conditionally lethal only^8^. For example, *Escherichia coli* strains lacking the functional *htrA* (*degP*) gene cannot survive at temperatures exceeding 40 °C^22^ or under certain conditions of oxidative stress^23^. *C*. *jejuni* Δ*htrA* strains are also sensitive to a combination of the oxidative and thermal stresses^24,25^. In *H*. *pylori*, in contrary to other bacterial species, we found that in only one of the 100 examined strains (N6) the *htrA* gene is dispensable for growth, which confirms the crucial role of *htrA* in this bacterium^16^. *H*. *pylori* lacking HtrA or with inactive HtrA exhibited reduced transmigration activity across polarized epithelial cells (Fig. 5). *H*. *pylori* binding to host cells was similar for the wild-type and mutant strains, while translocation of CagA by Δ*htrAsecA*R837K and Δ*htrA*/*htrA*S221A_N6_ mutants was significantly reduced in polarised Caco-2 cells in comparison to the wild-type or *htrA*-complemented strains (Fig. 6). Thus, we experimentally proved that HtrA is required for efficient *H*. *pylori* virulence connected with disruption of cell junctions. Successful construction of *htrA* mutant strains thus opens new research possibilities aiming at characterisation of the role of HtrA in *H*. *pylori* virulence and physiology (e.g., stress resistance).

However, deletion (N6 Δ*htrA*) or mutation of the *htrA* gene (N6 *htrA*S221A) selected for suppressor mutations in the *secA* gene. It was previously shown that the HtrA variants deprived of serine at the active site are proteolytically inactive, but they retain the chaperone activity^25,26^. Thus, the similar types of suppressor mutations (C852Y, C841Y, C843X, P858L or R837K) detected in N6 Δ*htrA* and N6 *htrA*S221A strains suggests that the proteolytic rather than chaperone activity of HtrA is essential for the *H*. *pylori* viability. Differences between *H*. *pylori* N6 and other strains, that allow the bacteria to survive without *htrA*, remain to be uncovered, but it should be noted that *H*. *pylori* is known for its high genetic variability between different species^27,28^. Thus, we suspected that *H*. *pylori* N6 may encode an additional protein (e.g. protease), which takes over the HtrA function in the *htrA* mutants. However, we couldn’t find any additional protease in N6 compared to other fully sequenced *H*. *pylori* genomes (Fig. S3). Alternatively, N6 may lack or produce fewer proteins, which may become toxic for *H*. *pylori* when accumulated in the periplasm of the Δ*htrA* or *htrA*S221A mutants. The latter hypothesis may be supported by the fact that *htrA* deletion or inactivation of the HtrA proteolytic activity induced mutations in the *secA* gene, thus possibly affected periplasmic homeostasis when compared to the wild-type cells.

The SecA protein is a partner of the bacterial SecYEG translocon that exports proteins to extracytoplasmic locations^29^. The system has been merely studied in *H*. *pylori* thus far^30–33^, although it is presumably the major protein translocation system in this bacterium^34,35^. Moreover, the Sec translocon is involved in translocation of vacuolating cytotoxin A (VacA) – one other major *H*. *pylori* virulence factor^36^. Inhibition of SecA synthesis lowers secretion of VacA, thus SecA is indirectly involved in *H*. *pylori* pathogenesis^30–32^.

SecA performs a dual role in protein translocation: (1) acts as an ATP-dependent motor to move a given polypeptide across the membrane, (2) participates in recruitment and delivery of suitable substrates to the Sec transmembrane channel. SecA is involved in the post-translational pathway of secretion and its substrates are primarily periplasmic and outer membrane proteins. Substrates can be bound co-translationally directly by SecA or they are delivered by the SecB chaperone to SecA (reviewed in^37^). SecA is a large protein and it is composed of several domains^38^. All substitutions found in the *secA* gene in the *ΔhtrA* or *htrA*S221A background mapped to the 5′ region coding for the C-terminal domain, including a zinc-binding motif (Fig. 3). Mutations of cysteine residues, C852Y and C841Y, retarded *H*. *pylori* growth; these mutants also exhibited lower *secA* expression, possibly further reducing SecA activity in *H*. *pylori* cells (Fig. 4). Interestingly, in another bacterium, *Acinetobacter baumanii*, truncation of the C-terminal part of SecA resulted in a reduction of the *secA* transcript level (to 69% of that detected in the wt parental bacteria)^39^. Moreover, a model SecA protein from *E*. *coli* is known to negatively regulate its own translation by binding to its own RNA and blocking the ribosome binding site^40^. Therefore, we may expect that also *H*. *pylori* SecA controls its own cellular content, possibly at translation step, and certain disturbances in the C-terminal part of this protein may affect this process. However, we also detected *secA* mutations (R837K and P857L), which neither changed *H*. *pylori* growth nor *secA* expression. Thus, the molecular mechanism of growth retardation and reduction of *secA* expression remains to be discovered. Nonetheless, the question arises why *htrA* inactivation selects for specific mutations in C-terminal domain of SecA?

The exact role of this region in SecA is not fully understood to date. It is known that C-terminus of *E*. *coli* SecA, in particular zinc binding domain, is responsible for specific interactions with SecB^21,41^. However, it is important to note that this domain is well conserved also in bacteria lacking SecB chaperone (e.g., *Bacillus subtilis* or *H*. *pylori*) (Fig. 5C and^33,42^). Moreover, SecB is not required for viability of *E*. *coli*^43^, while a lack of functional SecA is conditionally lethal in these bacteria. The importance of the very C-terminal 70–75 amino acids in the functionality of the SecA is a subject of controversy. In the work of Breukink *et al*.^44^ it was shown that SecA lacking its C-terminal 70 amino acids was not able to suppress the lethality of the temperature sensitive *secA* mutation (*secA51*(*Ts*) or *secA* amber) at the non-permissive temperature. Na *et al*.^45^ demonstrated that the C-terminal 70–75 amino acids can be deleted from SecA without losing complementation activity in *E*. *coli*. This domain of *E*. *coli* SecA contains a zinc binding motif mentioned above, composed of three cysteines and one histidine residue. In the presence of Zn^2+^, the fragment adopts a zinc finger-like motif^46^. The C-terminal part is also implicated in interaction with the inner membrane (acidic phospholipids) and was proposed to play an autoregulatory role. The C-terminally truncated variants showed an increased ATPase activity *in vitro*^44^. It has been also suggested that the C-terminal domain could auto-inhibit SecA by competing for interaction with substrate proteins but the significance of this activity is not clear^47^.

Whatever function is played by the C-terminal part of SecA, its alterations resulted in poor growth and a protein secretion defect in *E*. *coli*. These included C-terminal protein truncation^45^ or cysteine to serine substitutions^48^. Hence, by analogy, we can hypothesize that the *secA* suppressor mutations of Δ*htrA* or *htrA*S221A in *H*. *pylori* may lead to slowing down general protein translocation and, in consequence, lowering extracytoplasmic folding stress due to the absence or the lack of HtrA proteolytic activity. If this is the case, *secA* mutations in *H*. *pylori* would fall into place with suppression mutations detected in *htrA* mutants of other bacterial species. For example, in *E*. *coli* the majority of the compensatory (suppressor) mutations of the *htrA* temperature-sensitive phenotype lead to reduction of the envelope stress by either removal/release of HtrA substrates^49–52^ or by lowering synthesis of proteins that in the absence of HtrA may exert a proteotoxic effect on a bacterial cell^51,53^. Further studies are required to reveal the functional relationship between Sec translocon and HtrA in maintaining periplasm homeostasis. Interestingly, the cooperation of SecA and HtrA has been already suggested in *S*. *pneumonia* strain D39^7^, in which, during exponential growth and cell division, these two proteins co-localize.

In summary, we constructed the first *H*. *pylori* Δ*htrA* and *htrA*S221A mutant strains, which proved to be extremely valuable tools to study the role of HtrA in *H*. *pylori* virulence as demonstrated by the transmigration and CagA translocation assays. The suppressor mutations in *secA* detected in the *htrA* mutants suggest that lethality of *htrA* deletion/inactivation is caused by disturbed periplasmic homeostasis. The similar types of suppressor mutations coexisting with the *htrA* deletion or HtrA proteolytic activity inactivation indicates that the proteolytic rather than chaperone activity is essential for *H*. *pylori* survival. Hence, our results pinpointing a potential functional relationship between HtrA and the Sec translocon in *H*. *pylori*, together with previously reported cooperating between SecA and HtrA in *S*. *pneumonia*, possibly indicate more general mechanism responsible to maintain bacterial periplasmic homeostasis.

## Methods

### Materials, strains and culture conditions

All *H*. *pylori* strains tested in *htrA* gene mutagenesis are listed in the Supplementary Table S1, while *H*. *pylori htrA* mutants are listed in the Supplementary Table S2. The plasmids and *E*. *coli* bacterial strains used in this work are listed in the Supplementary Table S3. The oligonucleotide sequences are presented in the Supplementary Table S4. *E*. *coli* was grown at 37 °C on solid or liquid Luria-Bertani medium, supplemented with 50 µg/ml kanamycin where necessary. *E*. *coli* DH5α was used for cloning, while *E*. *coli* MC1061 was used for propagation of plasmids used to transform *H*. *pylori*. *H*. *pylori* was cultivated as described previously^54^ (for mutagenesis, DNA isolation or lysate preparation) or^17^ (for transwell migration studies). The liquid cultures were prepared in Brucella broth containing 10% fetal calf serum and antibiotic mix^55^. The growth of liquid cultures was monitored by measuring the optical density at 600 nm (OD_600_). Liquid cultures were set up by scraping *H*. *pylori* from blood agar plates by a sterile cotton swab and inoculating bacteria into Brucella broth pre-warmed to 37 °C to OD_600_~0.5 (OD_600_ = 1 corresponds to 1.4 × 10^9^ CFU/ml). *H*. *pylori* cells were grown for approx. 12 hours and then sub-cultured to a fresh medium pre-warmed to 37 °C to OD_600_ = 0.005. *H*. *pylori* culture grown for 40–45 hours, while OD_600_ of the culture was measured every 3–4 hours in the period of approx. 13–26 hours of growth. Growth rate G was calculated using formula G = t/((log(N/N_0_))/log^2^) (t, time of growth; N_0_, number of bacteria at the beginning of the time interval, N, number of bacteria at the end of the time interval). *H*. *pylori* cells were transformed with purified plasmid DNA by natural transformation^56^. For selection of the *H*. *pylori* transformants, kanamycin (15 µg/ml) or chloramphenicol (8 µg/ml) were added to the medium.

### *In vivo H. pylori* mutagenesis

#### N6 Δ*htrA* and N6 *htrA*S221A

The *H*. *pylori* 26695 genomic DNA served as a template in PCR reactions performed to prepare plasmids used to construct the mutants with chromosomal deletion of *htrA* or the mutation of *htrA* (661T > G) resulting in the synthesis of proteolytically inactive HtrAS221A. The regions flanking *htrA* were amplified by PCR using two pairs of primers complementary to the upstream (*rocE* fragment) and downstream (*ispDF* fragment) regions of *htrA*: H1-H2 and H3-H4, respectively. The resulting fragments and the non-polar *aphA*-*3* kanamycin resistance gene cassette^57^, cut out from the pILL2283 plasmid using BamHI-PstI restriction enzymes, were amplified by H1-H4 primer pair, digested by NdeI-EcoRI restriction enzymes and inserted into the NdeI-EcoRI sites of the pUC18 vector giving rise to plasmid pUZ16 (S3 Table). The pUZ16 plasmid was used to delete *htrA* from its native locus or it was further used as a cloning vector to prepare pUZ17.

To prepare pUZ17, *htrA* was amplified using H12-H13 primer pair and cloned into pUC19 digested by SmaI, giving pHJS3. The pHJS3 plasmid was used as a template to replace the codon of serine S221 with alanine by site-directed mutagenesis according to the standard protocol of the Quick- Change Mutagenesis Kit (Agilent) using H14- H15 primer pair and giving pUZ1. In parallel, *htrA* was amplified by PCR using H6-H7 primer pair. The PCR product was digested by NdeI-BamHI and cloned into pUZ16 digested by the same restriction enzymes giving pUZ18. Subsequently, by standard cloning, the SphI - AgeI fragment of *htrA* was exchanged for homologous *htrA* fragment containing 661T > G mutation, excised from pUZ1 by the same restriction enzymes, giving the final pUZ17 vector. The presence of 661T > G mutation introduced the PdiI restriction site into *htrA*, thus PdiI digestion was used to distinguish pUZ18 from pUZ17. *H*. *pylori* N6 was transformed with the pUZ16 or pUZ17 and plated on the BA plates supplemented with kanamycin. The colonies resistant to kanamycin were isolated and then analyzed by PCR, Southern blot, DNA sequencing, western blot, and casein zymography to prove that the proper *H*. *pylori htrA* deletion mutant (N6 Δ*htrA*) or the mutant synthesizing HtrAS221A mutant protein (N6 *htrA*S221A) were obtained.

#### N6 Δ*htrA*/*htrA*_N6_

A three-step strategy was used to generate a complementation strain of the *H*. *pylori htrA* deletion mutant strain, that contained the re-introduced wild-type *htrA* gene in its native position within the genome (Fig. S4). First, the three DNA fragments were amplified using the following primer pairs: H1-H16 for *rocE*-*htrA*, H17-H4 for *ispDF* and H18-H19 for chloramphenicol cassette (*cat*). The *H*. *pylori* N6 wild-type genomic DNA served as a template in PCR for the *rocE*-*htrA* and *ispDF* fragments, while pILL2150 served as a template for the *cat* cassette. Next, the resulting fragments were amplified by H1-H4 primer pair and the full-length fusion PCR product (approximately 0.8 µg) was used to transform the *H*. *pylori* N6 Δ*htrAsecA*R837K strain (Table S2). After antibiotic selection, correct integration of the *htrA* gene was verified by sequencing. Expression of the HtrA protein and its ability to digest casein was checked in the selected *H*. *pylori* N6 Δ*htrA*/*htrA*_N6_ clones using western blotting and zymography, respectively.

#### N6 Δ*htrA*/*htrA*S221A_N6_

A multistep strategy was used to generate a complementation strain of the *H*. *pylori htrA* deletion mutant strain, that contained the re-introduced mutated *htrA* 661T > G gene in its native position within the genome (Fig. S6). First, pUZN10 and pUZN11 were generated to prepare a mutated version of *H*. *pylori* N6*htrA* 661T > G. *htrA* was amplified using H22-H23 primer pair and *H*. *pylori* N6 genomic DNA, cloned into pET26b digested by NcoI-XhoI, giving pUZN10. The pUZN10 plasmid was used as a template to replace the codon of serine S221 with alanine by site-directed mutagenesis according to the standard protocol of the SapphireAmp Fast polymerase (Takara) using H24- H25 primer pair and giving pUZN11. Next, the three DNA fragments were amplified using the following primer pairs: H1-H21 for *rocE*-*htrA*, H20-H16 for *htrA* 661G > T, and H18-H4 for *cat*-*ispDF*. The *H*. *pylori* N6 wild-type genomic DNA served as a template in PCR for the *rocE*-*htrA*, N6 Δ*htrA*/*htrA*_N6_ for *cat*-*ispDF*, while pUZN11 served as a template for the *htrA* 661T > G. Next, the resulting fragments were amplified by H1-H4 primer pair and the full-length fusion PCR product (approximately 0.8 µg) was used to transform the *H*. *pylori* N6 Δ*htrAsecA*R837K strain (Table S2). Selection and analysis of *H*. *pylori* N6 Δ*htrA*/*htrA*S221A_N6_ clones were done as described for N6 Δ*htrA*/*htrA*_N6_.

#### N6 Δ*cagY*

*H*. *pylori* mutant deficient in the *cagY* (*virB10*) gene of the type IV secretion system was constructed as described^58^.

### Southern blot

Southern blot was performed as described^59^. Briefly, 10 μg of *H*. *pylori* genomic DNA and 10 ng of a control plasmid DNA isolated from *E*. *coli*, digested with HindIII, were resolved in 1% agarose gel. DNA was transferred onto a nylon membrane and incubated at 68 °C with digoxigenin-labeled DNA probe (431 bp DNA, amplified with primers H8-H9). Southern blot was developed by a colorimetric reaction using anti-digoxygenin antibody (Anti-Digoxigenin-AP, Fab fragments, Roche).

### SDS-PAGE, western blotting and Casein zymography

SDS-PAGE and western blotting were described elsewhere^59,60^. For western blotting, proteins were transferred onto PVDF membranes (Immobilon-P, Merck Millipore). All steps were carried in TBS-T buffer (140 mM NaCl, 25 mM Tris- HCl, pH 7.4, 0.1% Tween- 20). The membranes were blocked with 5% non-fat milk or 3% BSA (for detection of phosphorylated CagA). Non-phosphorylated and phosphorylated CagA protein species were detected using the rabbit polyclonal α‐CagA antibody (# HPP‐5003‐9, Austral Biologicals, San Ramon/USA) and α-PY‐99 antibody as described^61^. A monoclonal mouse antibody against GAPDH (# sc‐20357, Santa Cruz) expression was applied as a loading control. HtrA was detected by using polyclonal rabbit anti-HtrA antibodies followed by the secondary goat anti-rabbit polyvalent, horseradish peroxidase-conjugated IgGs (catalogue number #31462, Life Technologies, Darmstadt/Germany). Anti-HtrA antibodies were raised in rabbit against the purified recombinant *H*. *pylori* HtrA. Experimental procedures were conducted according to the Interdisciplinary Principles and Guidelines for the Use of Animals in Research, Marketing and Education issued by the New York Academy of Sciences’ Ad Hoc Committee on Animal Research, and Directive 2010/63/UE of the European Parliament and of the Council of 22 September 2010 on the protection of animals used for scientific purposes. All experiments were approved by the First Local Committee for Experiments with the Use of Laboratory Animals, Wroclaw, Poland (permission number 50/2015). Casein zymography was performed as described^16^.

### Human cell culture

The non-polarized human gastric adenocarcinoma cell line AGS (ATCC CRL‐1739™) was cultured in RPMI 1640 medium containing 2 mM L‐glutamine (Invitrogen, Karlsruhe/Germany) and 10% heat‐inactivated fetal calf serum (FCS; Gibco, Paisley/UK). Caco-2 (ATCC HTB‐37) represents a polarized human colon adenocarcinoma cell line and was cultured in Dulbecco’s Modified Eagle Medium (DMEM) supplemented by 110 mg/L sodium pyruvate, 4.5 g/L D‐glucose, 4 mM L‐glutamine and 10% FCS. The human cell line MKN28 was kindly provided by Motomo Kuroki (Fukuoka University/Japan) and is phenotypically different from the MKN28 cell line available from the JCRB cell bank (number 0253) described previously^14,62^. These cells were cultured in Eagle’s minimum essential medium (Sigma‐Aldrich) with 10% FCS. All cell lines were generally supplemented with 1% antibiotic and antimycotic solution (Sigma‐Aldrich) and grown in incubators with 5% (v/v) CO_2_ at 37 °C. Subculturing was performed at a ratio of 1:3 to 1:5 at a confluence of 70 to 90% every 2 to 3 days. Every cell line was cultured in 75 cm^2^ tissue culture flasks and subculturing in 6-well plates (Greiner‐Bio‐One, Germany), and washed with antibiotics-free medium before infection.

### Cell binding and elongation assay

Infection of non-polarized AGS cells (80% confluence) and confluent polarized Caco-2 monolayers was performed in triplicates on six‐well plates^63^. After 6-hour co-incubation with the different indicated *H*. *pylori* strains at MOI of 25, infected cells were rigorously washed three times using 1 mL of pre-warmed culture medium (without antibiotics) to remove non-bound *H*. *pylori*. For quantification of cell‐bound bacteria as colony forming units (CFU), the cells were incubated with1 mL of buffer (PBS containing 0.1% saponin) for 15 min at 37 °C as described^64^. The resulting suspensions were diluted in serial steps and incubated on GC agar plates for 5 days, followed by quantification of the number of CFUs. The number of elongated cells after infection was done as described previously^65^. For western blotting cells were collected as described^61^.

### Transwell infection studies

Transwell infection studies were done as described earlier^17^. For this purpose, the cells were cultured on 0.33 cm^2^ cell culture inserts (with 3 μm pore size). The cells were grown to confluent monolayers and then incubated for another 14 days to allow cell polarization. The cells were infected in the apical compartment at MOI of 50 in a time course and the numbers of transmigrated bacteria were quantified in aliquots taken from the basal chambers and counting CFUs on GC agar plates after 5 days of incubation.

### DNA Sequencing

Sanger sequencing of DNA fragments was performed by Genomed SA (Poland) or GATC Biotech (Germany). Next generation sequencing was performed by Biobank Lab, University of Lodz (Poland) and FAU Erlangen (Germany). The genome of *H*. *pylori* N6 wt was sequenced on Illumina NexSeq. 500 (Lodz), Illumina MiSeq (Germany) and ONT (Oxford Nanopore Technologies) MinION single molecule sequencing platform (Lodz). The genomes of *H*. *pylori* Δ*htrA* and *htrA*S221A strains were sequenced on Illumina NextSeq500 (Lodz) and Illumina MiSeq (Germany). Short reads libraries for all samples were prepared with the Nextera XT kit, in accordance with the manufacturer’s instruction (Illumina, San Diego, USA). Libraries for ONT MinION platform were prepared with ONT Rapid Sequencing Kit (SQK-RAD004, Oxford, UK). Illumina sequencing was performed at a reads length 2 × 150 bp for NextSeq platform and 2 × 300 bp for MiSeq.

### qRT-PCR

RNA was extracted from three independent sets of *H*. *pylori* cultures in the logarithmic phase of growth (OD_600_ ~ 0.4–0.7). RNA was extracted using a Total RNA Extraction Plus kit (A&A Biotechnology) according to the manufacturer’s protocol, and further treated with RNase-free DNAseI (Thermo Scientific). Reverse transcription (RT) reactions were carried out on 0.5 μg of total RNA in 20 μl using Maxima Reverse Transcriptase (Thermo Scientific) and random hexamer primers in the presence of RiboLock RNase Inhibitor (Thermo Scientific), as described by the manufacturer. mRNA levels of the selected *H*. *pylori* genes were quantified by qPCR, performed on a CFX96 Touch Real-Time PCR Detection System (BioRad) using SensiFAST SYBR No-ROX (BioLine) and the following parameters: 96 °C for 2 min, followed by 40 three-step amplification cycles consisting of 5 s at 96 °C, 10 s at 60 °C and 10 s at 72 °C. Reaction mixtures (15 µl) contained qPCR mix (7.5 μl), cDNA (1 μl of 50x diluted RT reaction) and primers (0.3 µM each). The following primer pairs were used: *secA*, H28-H29; *nifS*, H30-H31 and 16SrRNA, H32-H33. The relative quantity of mRNA for each gene was determined by reference to the mRNA levels of *H*. *pylori* 16SrRNA.

### *In silico* analysis

Illumina raw reads were quality and length trimmed with Trim Galore! v. 0.4.5 (https://www.bioinformatics.babraham.ac.uk/projects/trim_galore/). Trimmed data from Illumina and Nanopore sequencing of *H*. *pylori* N6 wild-type strain were used to prepare high contiguity reference genome of starting strain. *De novo* assembly of *H*. *pylori* N6wt was prepared with SPAdes 3.10^66^ hybrid assembly option. Gene annotation was done with Prokka v. 1.12^67^. Reads mapping and variant calling versus earlier prepared reference genome of *H*. *pylori* N6wt was performed with CLC Genomics Workbench v. 8.5.1 and Snippy v. 3.2 (Snippy 9, https://github.com/tseemann/snippy). The massive deletion was identified by visualization mapped reads in IGV v. 2.4.0^68^ and analysis of the obtained image.

### Equipment and settings

DNA/protein electrophoresis and chemiluminescent western blot results were documented by the Gel Doc™ XR+ System or ChemiDoc XRS+ and processed by Image Lab software. RT-qPCR was performed using CFX96 Real-Time System and CFX manager software. The images were prepared for publication by CorelDRAW and CorelPHOTO-PAINT software. Digital processing was applied equally across the entire image, including controls.

### Statistical data analysis

Each experiment was performed independently for at least three times with similar results. The data were evaluated using the Student’s t‐test with SigmaPlot statistical software (version 13.0) or Excell 2016.

## Supplementary information

Supplementary Files

## Data Availability

This Whole Genome Shotgun projects (Bioproject PRJNA505142) have been deposited at DDBJ/ENA/GenBank under the accession numbers: SAMN10411099 (N6 wild-type strain), SAMN10411397 (N6 Δ*htrA*) and SAMN10411398 (N6 *htrA*S221A). The version described in this paper is version SAMN01000000.
