# Supplementary material for: Establishment of serine protease htrA mutants in Helicobacter pylori is associated with secA mutations
Source: Sci Rep. 2019 Aug 13;9:11794. doi: 10.1038/s41598-019-48030-6 (PMC6692382; doi:10.1038/s41598-019-48030-6)
Supplement: Supplementary file 1 — Supplementary Files [file 41598_2019_48030_MOESM1_ESM.pdf]

Supplementary Information

"Establishment of serine protease *htrA* mutants in *Helicobacter pylori* is associated with *secA* mutations"

Anna Zawilak-Pawlik[1*], Urszula Zarzecka[2,3], Dorota Żyła-Uklejewicz[1], Jakub Lach[4,5], Dominik Strapagiel[4,5], Nicole Tegtmeyer[3], Manja Böhm[3], Steffen Backert[3], Joanna Skorko-Glonek[2*]

[1] Hirszfeld Institute of Immunology and Experimental Therapy, Polish Academy of Sciences, Department of Microbiology, Weigla 12, 53-114 Wrocław, Poland;

[2] Department of General and Medical Biochemistry, Faculty of Biology, University of Gdańsk, Wita Stwosza 59, 80-308 Gdańsk, Poland.

[3] Department of Biology, Division of Microbiology, Friedrich-Alexander University Erlangen-Nuremberg, Staudtstr. 5, 91058 Erlangen, Germany

[4] BBMRI.pl Consortium, Stabłowicka 147, 54-066 Wrocław, Poland

[5] Biobank Lab, Department of Molecular Biophysics, Faculty of Biology and Environmental Protection, University Lodz, Pilarskiego 14, Lodz, Poland

[*]Correspondence:

Anna Zawilak-Pawlik Tel.: +48713709949; Fax: +48713371382; Email: anna.pawlik@hirszfeld.pl

Joanna Skorko-Glonek Tel.: +48585236056 ; Fax: +48585236064; Email: joanna.skorko-glonek@biol.ug.edu.pl

S1 Table. *H. pylori* strains tested in *htrA* gene mutagenesis.

Number	*H. pylori* strain	Origin	Reference	HtrA deletion[a]	HtrA mutation[b]
1	N6	France	[1]	+	+
2	X47 AL	France	[1]	-	-
3	26695	UK	[2]	-	-
4	J99	USA	[3]	-	-
5	Sat464	Peru	[4]	-	-
6	Lit75	Lithuania	[4]	-	-
7	Shi470	Peru	[4]	-	-
8	Cuz20	Peru	[4]	-	-
9	Pecan4	Peru	[4]	-	-
10	SJM180	Peru	[4]	-	-
11	India7	India	[4]	-	-
12	Safr7	South Africa	[5]	-	-
13	B128	USA	[6]	-	-
14	7.13	USA	[6]	-	-
15	B8	USA	[7]	-	-
16	Gam94/24	Gambia	[4]	-	-
17	G27	Italy	[8]	-	-
18	HPAG1	Sweden	[9]	-	-
19	TN2-GF4	Japan	[10]	-	-
20	83	Japan	[11]	-	-
21	35A	Japan	[11]	-	-
22	B38	France	[12]	-	-
23	1061	Netherlands	[13]	-	-
24	P1	Germany	[14]	-	-
25	P12	Germany	[15]	-	-
26	P49	Germany	[16]	-	-
27	P76	Germany	[17]	-	-
28	P227	Germany	[18]	-	-
29	P282	Germany	[19]	-	-
30	P284	Germany	[20]	-	-
31	P300	Germany	[21]	-	-
32	P303	Germany	[20]	-	-
33	P310	Germany	[22]	-	-
34	P340	Germany	[23]	-	-
35	P1280	Germany	[20]	-	-
36	P1288	Germany	[20]	-	-
37	P1303	Germany	[20]	-	-
38	P1321	Germany	[20]	-	-
39	2808	Germany	[22]	-	-
40	11637	Japan	[22]	-	-

41	Ka88	Germany	[22]	-	-
42	Ka204	Germany	[22]	-	-
43	Hel230	Sweden	[24]	-	-
44	Hel305	Sweden	[24]	-	-
45	Hel312	Sweden	[25]	-	-
46	Hel333	Sweden	[25]	-	-
47	2003-370	Mexico	[23]	-	-
48	HP77	Greece	[23]	-	-
49	11638	Australia	[23]	-	-
50	USA2964	USA	[23]	-	-
51	3461	Japan	[23]	-	-
52	Oki105	Japan	[23]	-	-
53	Oki149	Japan	[23]	-	-
54	Oki326	Japan	[23]	-	-
55	Oki388	Japan	[23]	-	-
56	Oki61	Japan	[26]	-	-
57	Ind69	Indonesia	[27]	-	-
58	FD453	Malaysia	[27]	-	-
59	CH7	China	[27]	-	-
60	Mand38	Myanmar	[27]	-	-
61	2002-14	Mexico	[27]	-	-
62	PMSS1	Australia	[28]	-	-
63	SS1	Australia	[28]	-	-
64	147A	USA	[29]	-	-
65	147C	USA	[29]	-	-
66	Tx30	Germany	[30]	-	-
67	Ka89	Germany	[30]	-	-
68	CCUG17875	Sweden	[31]	-	-
69	17875/Leb	Sweden	[31]	-	-
70	UH4	Germany	[32]	-	-
71	UH44	Germany	[32]	-	-
72	Ka36	Germany	[32]	-	-
73	Ka44	Germany	[32]	-	-
74	Ka52	Germany	[32]	-	-
75	Ka89	Germany	[32]	-	-
76	Ka92	Germany	[32]	-	-
77	Ka125	Germany	[32]	-	-
78	Ka148/1	Germany	[32]	-	-
79	Ka148/2	Germany	[32]	-	-
80	Ka156	Germany	[32]	-	-
81	Ka161	Germany	[32]	-	-
82	Ka171	Germany	[32]	-	-
83	Ka223	Germany	[32]	-	-
84	Ca018	Germany	[32]	-	-
85	Ca070	Germany	[32]	-	-

86	Ca073	Germany	[32]	-	-
87	Ca088	Germany	[32]	-	-
88	Ca117	Germany	[32]	-	-
89	Ca130	Germany	[32]	-	-
90	Ca173	Germany	[32]	-	-
91	Ca202	Germany	[32]	-	-
92	Ca204	Germany	[32]	-	-
93	OM1011	Germany	[32]	-	-
94	OM12005	Germany	[32]	-	-
95	MPI47	Germany	[32]	-	-
96	M4	Germany	[32]	-	-
97	M55	Germany	[32]	-	-
98	HPK1	Japan	[33]	-	-
99	HPK5	Japan	[33]	-	-
100	84-183	USA	[33]	-	-

[a] *htrA* gene deletion mutagenesis as described in the Materials and Methods section. +, positive; -, negative.
[b] *htrA* gene inactivation by S221A point mutation as described in the Materials and Methods section. +, positive; -, negative.

S2 Table. *H. pylori htrA* mutant strains.

Strain	genotype	Reference/ source
N6 Δ*htrA*		
N6 Δ*htrAsecA*R837K (1)	*htrA::aphA-3, secA2510G>A*	This work
N6 Δ*htrAsecA*C852Y (2)	*htrA::aphA-3, secA2555G>A*	This work
N6 *htrAS221A*		
N6 *htrAS221secAC841Y* (2)	*htrA*::*htrA*661T>G-*aphA-3, secA2522G>A*	This work
N6 *htrAS221secAC843X* (3)	*htrA*::*htrA*661T>G-*aphA-3, secA2529T>A*	This work
N6 *htrAS221secAP858L* (4)	*htrA*::*htrA*661T>G-*aphA-3, secA2573C>T*	This work
N6 Δ*htrA/htrA*$_{N6}$	(*htrA::aph-3)::htrA*$_{N6}$-*cat, secA2510G>A*	This work
N6 Δ*htrA/htrA*S221A$_{N6}$	(*htrA::aph-3)::htrA661T>G*$_{N6}$-*cat, secA2510G>A*	This work
N6 Δ*cagY*	*cagY::cat*	This work

S3 Table. *E. coli* strains and recombinant plasmids.

Strain/ plasmid	genotype	Reference/ source
E. coli		
E. coli DH5α	supE44, hsdR17, recA1, endA1, gyrA1, gyrA96, thi-1, relA1	Laboratory stock
E. coli MC1061	F araD139(ara-leu)7696 galE15 galK16(lacX74) rps hsdR^{2}mcrA mcrB1	[34]
plasmids		
pUC18	Cloning vector, Amp^{R}	MBI Fermentas
pUC19	Cloning vector, Amp^{R}	MBI Fermentas

pILL2283	pUC18 derivative containing upstream and downstream *hobA* flanking regions separated by a non-polar *aphA-3*, used as a source of *aphA*-3 cassette, Amp^R Kan^R	[35]
pILL2150	*E. coli-H. pylori* shuttle, inducible expression vector	[36]
pUZ1	pUC19 derivative containing *htrA* (26695 strain) mutated at 661T>G, Amp^R	This work
pUZN10	pET26b derivative containing *htrA* (N6 strain) expressed in fusion with C-terminal 6xHis-tag, Kan^R	This work
pUZN11	pET26b derivative containing *htrA* (N6 strain) mutated at 661T>G, expressed in fusion with C-terminal 6xHis-tag, Kan^R	This work
pUZ16	pUC18 derivative, containing upstream (*rocE*) and downstream (*ispDF*) *htrA* flanking regions separated by a non-polar *aphA-3*, used for *htrA* deletion by allelic exchange, Amp^R Kan^R	This work
pUZ17	pUC18 derivative containing *htrA* (26695 strain) mutated at 661T>G and the *htrA* downstream region separated by a non-polar *aphA-3*, used for introduction of 661T>G into *htrA* by allelic exchange, Amp^R Kan^R	This work
pUZ18	pUZ16 derivative, in which the upstream region of *htrA* (*rocE*) was replaced by the full length *htrA* gene	This work
pHJS3	pUC19 derivative containing wild type *htrA* (26695 strain), Amp^R	This work

S4 Table. Primers used in this studies.

name	Sequence (5' → 3')
H1	GAACATATGTCGTGGAAGCTTTGATTAATG
H2	CATTTATTCCTCCTAGTTAGTCAGGATCCCATGGTTACTTCCTTAAAATTTGCTA
H3	TAGTACCTGGAGGGAATAATGCTGCAGATGTCTTTGATTAGAGTGAATGGGG
H4	CGGAATTCCAAGCTTGATTTCAGGGTAATGG
H6	GAACATATGAAAAAAACCCTTTTTATCTCTTTG
H7	CGGGATCCTCATTTCACCAAAATGATCCTATAAC
H8	TCCACTGAAAAGAAGATTAAAAAC
H9	CCAATCCCGCTTTTATTGAGC
H10	GCAAGATTCTAGCGATGCGG

H11	GCTTGTGGTATAGAGCGTTAAGG
H12	ATACTCGAGTTTCACCAAAATGATTCTATAACCTTG
H13	ATACCATGGGCAATATCCAAATCCAGAGCATGCCC
H14	CCTGGAAATGCCGGCGGCGCTTTAATTGATAGC
H15	GCTATCAATTAAAGCGCCGCCGGCATTTCCAGG
H16	TTTTCTCCATTTTAGCTTCCTAGTTAGTCATTTCACCAAAATGATCC
H17	GAGTGGCAGGGCGGGGCGTAATACCTGGAGGGAATAATGCTG
H18	GGATCATTTTGGTGAAATGACTAACTAGGAAGCTAAAATGGAGAAAA
H19	CAGCATTATTCCCTCCAGGTATTACGCCCCGCCCTGCCACTC
H20	CTTTAGCGTTAAGCTTGAATGCGGGCAATATCCAAATCCAG
H21	CTGGATTTGGATATTGCCCGCATTCAAGCTTAACGCTAAAG
H22	ATACCATGGGCAATATCCAAATCCAAAGC
H23	ATACTCGAGTTTCACCAAAATGATCC
H24	CCTGGAAATGCCGGCGGCGCTTTGATTGATAGC
H25	GCTATCAATCAAAGCGCCGCCGGCATTTCCAGG
H26	CCACTTATGCGATGTTTTTTTCCC
H27	TTTTTGAGCGCTTCAATATTGGC
H28	TGAAAGACATGAGAGCCGCA
H29	CCCCTTAATCCTGTCGCTCC
H30	AAGGGATCATTACGGGAACC
H31	GTGGGTTTCTGTGCCAAACT
H32	CTAGCGGATTCTCTCAATGTCAA
H33	GGAGTACGGTCGCAAGATTAAA

References

1. Ferrero, R. L., Cussac, V., Courcoux, P. & Labigne, A. Construction of isogenic urease-negative mutants of *Helicobacter pylori* by allelic exchange. *J. Bacteriol.* **174,** 4212–4217 (1992).
2. Tomb, J. F. *et al.* The complete genome sequence of the gastric pathogen *Helicobacter pylori*. *Nature* **388,** 539–547 (1997).
3. Alm, R. A. *et al.* Genomic-sequence comparison of two unrelated isolates of the human gastric pathogen *Helicobacter pylori*. *Nature* **397,** 176–180 (1999).
4. Kersulyte, D. *et al. Helicobacter pylori* from Peruvian Amerindians: Traces of Human Migrations in Strains from Remote Amazon, and Genome Sequence of an Amerind Strain. *PLoS One* **5,** e15076 (2010).
5. Duncan, S. S. *et al.* Genome Sequences of Three hpAfrica2 Strains of *Helicobacter pylori*. *Genome Announc.* **1,** e00729-13 (2013).
6. Franco, A. T. *et al.* Delineation of a carcinogenic *Helicobacter pylori* proteome. *Mol. Cell. Proteomics* **8,** 1947–58 (2009).
7. Farnbacher, M. *et al.* Sequencing, annotation, and comparative genome analysis of the gerbil-adapted *Helicobacter pylori* strain B8. *BMC Genomics* **11,** 335 (2010).
8. Baltrus, D. A. *et al.* The complete genome sequence of Helicobacter pylori strain G27. *J. Bacteriol.* **191,** 447–8 (2009).
9. Oh, J. D. *et al.* The complete genome sequence of a chronic atrophic gastritis *Helicobacter pylori* strain: evolution during disease progression. *Proc. Natl. Acad. Sci. U.*

S. A. **103,** 9999–10004 (2006).

10. Watanabe, T., Tada, M., Nagai, H., Sasaki, S. & Nakao, M. *Helicobacter pylori* infection induces gastric cancer in mongolian gerbils. *Gastroenterology* **115,** 642–8 (1998).
11. Yahara, K. *et al.* Chromosome Painting *In Silico* in a Bacterial Species Reveals Fine Population Structure. *Mol. Biol. Evol.* **30,** 1454–1464 (2013).
12. Thiberge, J.-M. *et al.* From array-based hybridization of *Helicobacter pylori* isolates to the complete genome sequence of an isolate associated with MALT lymphoma. *BMC Genomics* **11,** 368 (2010).
13. Backert, S. *et al.* Translocation of the *Helicobacter pylori* CagA protein in gastric epithelial cells by a type IV secretion apparatus. *Cell. Microbiol.* **2,** 155–64 (2000).
14. Moese, S. *et al.* Identification of a tyrosine-phosphorylated 35 kDa carboxy-terminal fragment (p35CagA) of the *Helicobacter pylori* CagA protein in phagocytic cells: Processing or breakage? *Proteomics* **1,** 618–629 (2001).
15. Fischer, W. *et al.* Strain-specific genes of *Helicobacter pylori*: genome evolution driven by a novel type IV secretion system and genomic island transfer. *Nucleic Acids Res.* **38,** 6089–6101 (2010).
16. Kwok, T., Backert, S., Schwarz, H., Berger, J. & Meyer, T. F. Specific entry of *Helicobacter pylori* into cultured gastric epithelial cells via a zipper-like mechanism. *Infect. Immun.* **70,** 2108–20 (2002).
17. Ramarao, N., Gray-Owen, S. D., Backert, S. & Meyer, T. F. *Helicobacter pylori* inhibits phagocytosis by professional phagocytes involving type IV secretion components. *Mol. Microbiol.* **37,** 1389–404 (2000).
18. Backert, S., Müller, E.-C., Jungblut, P. R. & Meyer, T. F. Tyrosine phosphorylation patterns and size modification of the *Helicobacter pylori* CagA protein after translocation into gastric epithelial cells. *Proteomics* **1,** 608–617 (2001).
19. Selbach, M. *et al.* The *Helicobacter pylori* CagA protein induces cortactin dephosphorylation and actin rearrangement by c-Src inactivation. *EMBO J.* **22,** 515–28 (2003).
20. Gieseler, S., König, B., König, W. & Backert, S. Strain-specific expression profiles of virulence genes in *Helicobacter pylori* during infection of gastric epithelial cells and granulocytes. *Microbes Infect.* **7,** 437–447 (2005).
21. Backert, S., Kwok, T. & König, W. Conjugative plasmid DNA transfer in *Helicobacter pylori* mediated by chromosomally encoded relaxase and TraG-like proteins. *Microbiology* **151,** 3493–3503 (2005).
22. Brandt, S., Kwok, T., Hartig, R., König, W. & Backert, S. NF-kappaB activation and potentiation of proinflammatory responses by the *Helicobacter pylori* CagA protein. *Proc. Natl. Acad. Sci. U. S. A.* **102,** 9300–5 (2005).
23. Mueller, D. *et al.* c-Src and c-Abl kinases control hierarchic phosphorylation and function of the CagA effector protein in Western and East Asian *Helicobacter pylori* strains. *J. Clin. Invest.* **122,** 1553–66 (2012).
24. Enarsson, K., Brisslert, M., Backert, S. & Quiding-Järbrink, M. *Helicobacter pylori* induces transendothelial migration of activated memory T cells. *Infect. Immun.* **73,** 761–9 (2005).
25. Brisslert, M. *et al. Helicobacter pylori* induce neutrophil transendothelial migration: role of the bacterial HP-NAP. *FEMS Microbiol. Lett.* **249,** 95–103 (2005).
26. Lind, J. *et al.* Systematic Analysis of Phosphotyrosine Antibodies Recognizing Single

Phosphorylated EPIYA-Motifs in CagA of Western-Type *Helicobacter pylori* Strains. *PLoS One* **9,** e96488 (2014).

27. Lind, J. *et al.* Systematic analysis of phosphotyrosine antibodies recognizing single phosphorylated EPIYA-motifs in CagA of East Asian-type *Helicobacter pylori* strains. *BMC Microbiol.* **16,** 201 (2016).
28. Lee, A. *et al.* A standardized mouse model of *Helicobacter pylori* infection: introducing the Sydney strain. *Gastroenterology* **112,** 1386–97 (1997).
29. Zhang, M. High antibiotic resistance rate: A difficult issue for *Helicobacter pylori* eradication treatment. *World J. Gastroenterol.* **21,** 13432–13437 (2015).
30. Javaheri, A. *et al. Helicobacter pylori* adhesin HopQ engages in a virulence-enhancing interaction with human CEACAMs. *Nat. Microbiol.* **2,** 16189 (2017).
31. Olofsson, A. *et al.* Biochemical and functional characterization of *Helicobacter pylori* vesicles. *Mol. Microbiol.* **77,** 1539–1555 (2010).
32. Backert, S. *et al.* Functional analysis of the cag pathogenicity island in *Helicobacter pylori* isolates from patients with gastritis, peptic ulcer, and gastric cancer. *Infect. Immun.* **72,** 1043–56 (2004).
33. Kuipers, E. J., Israel, D. A., Kusters, J. G. & Blaser, M. J. Evidence for a conjugation-like mechanism of DNA transfer in *Helicobacter pylor*i. *J. Bacteriol.* **180,** 2901–5 (1998).
34. Casadaban, M. J. & Cohen, S. N. Analysis of gene control signals by DNA fusion and cloning in *Escherichia coli*. *J. Mol. Biol.* **138,** 179–207 (1980).
35. Zawilak-Pawlik, A. *et al.* HobA--a novel protein involved in initiation of chromosomal replication in *Helicobacter pylori*. *Mol. Microbiol.* **65,** 979–994 (2007).
36. Boneca, I. G. *et al.* Development of inducible systems to engineer conditional mutants of essential genes of *Helicobacter pylori*. *Appl. Environ. Microbiol.* **74,** 2095–2102 (2008).

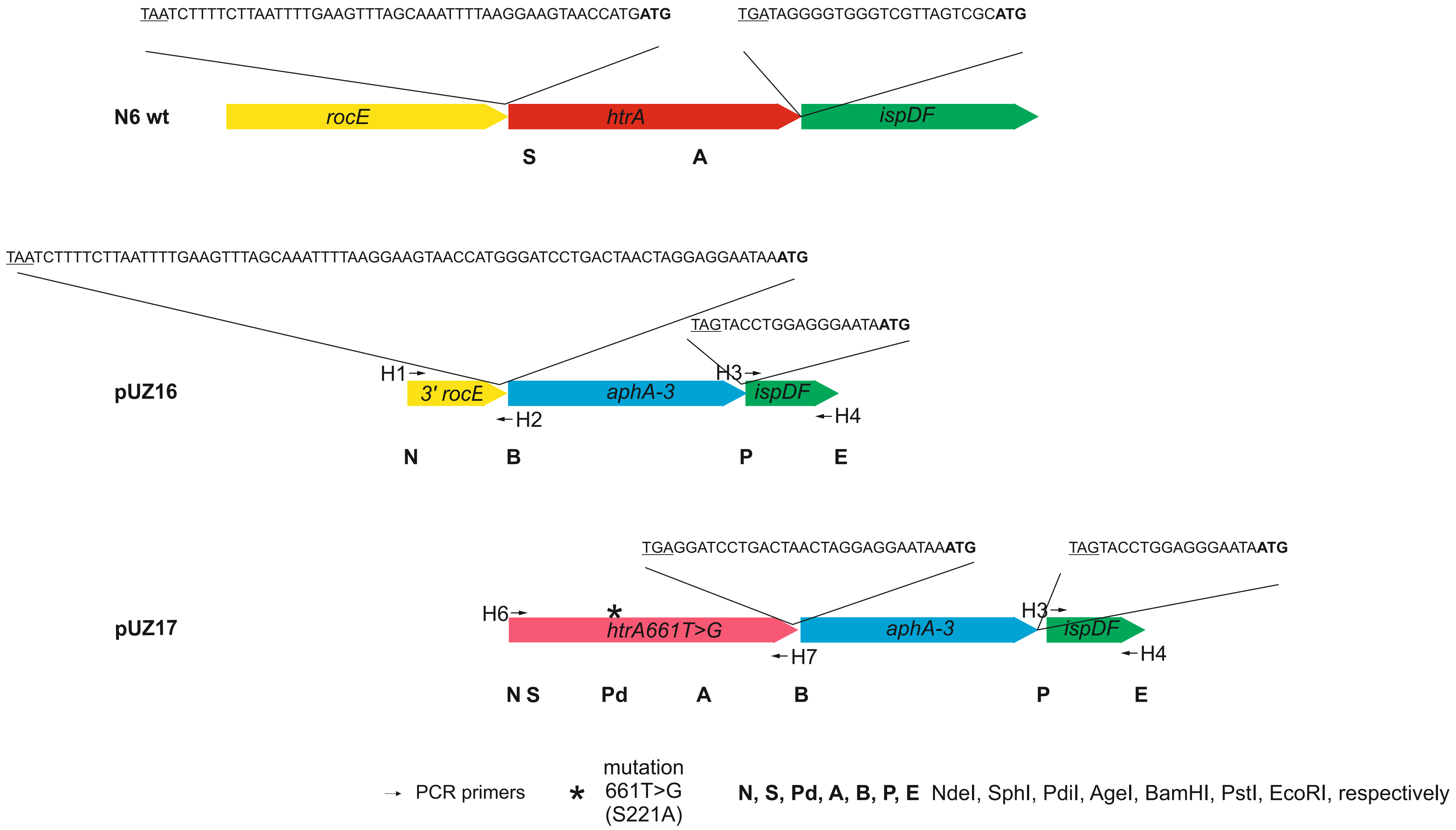

Fig. S1. The mutagenesis strategy used to delete or mutate *htrA* on the *H. pylori* chromosome. *H. pylori* N6 wild-type *htrA* chromosomal locus and plasmid-encoded DNA fragments, which recombined with *H. pylori* chromosome via double crossing over to give Δ*htrA* and *htrA*S221A mutant strains, are shown. Sequences of intergenic regions are presented; stop and start codons are underlined or presented in bold, respectively. Primer sequences are given in S4 Table.

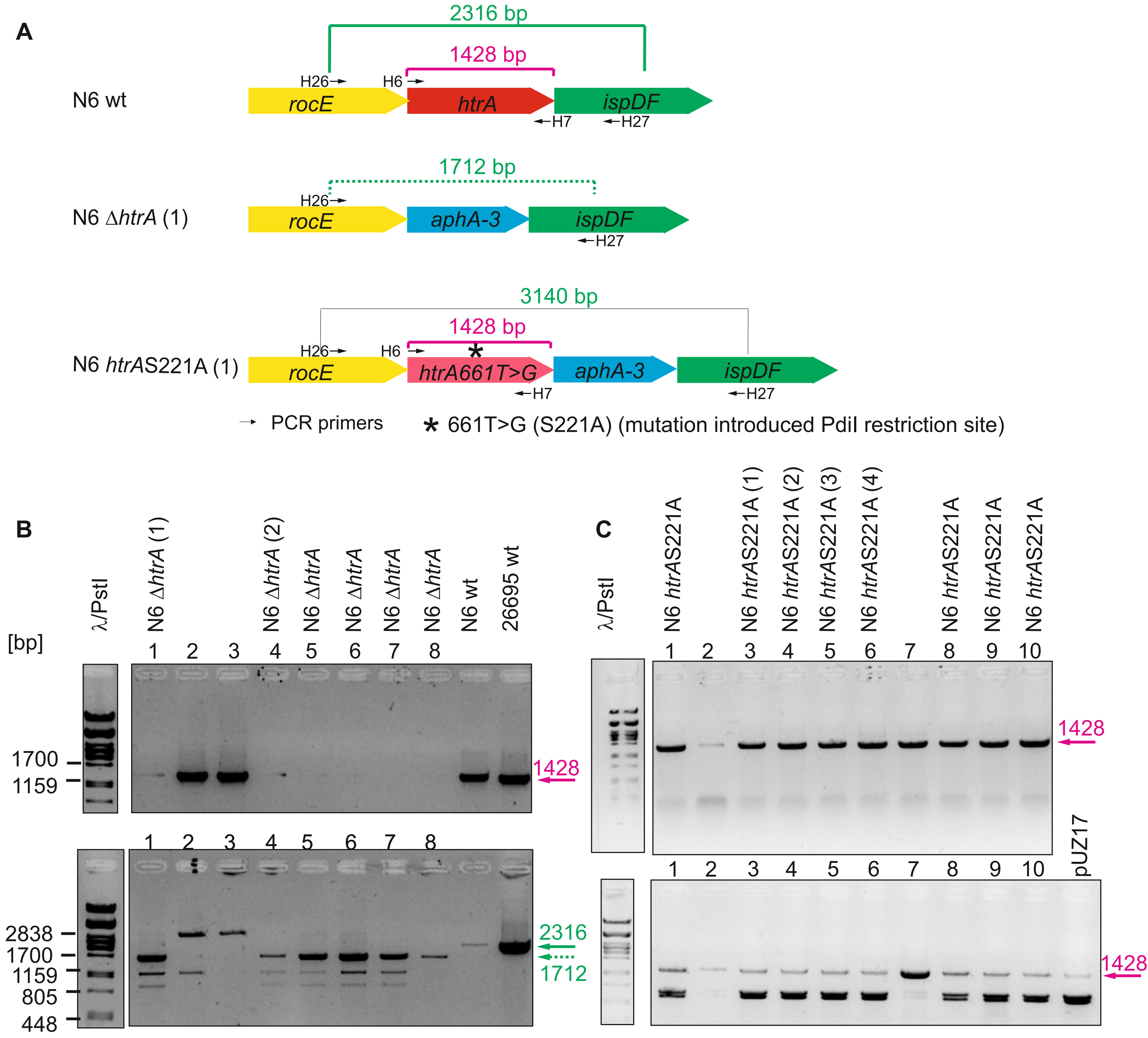

Fig. S2. Analysis of *H. pylori* Δ*htrA* and *htrA*S221A mutated strains. Numbers in brackets denotes independent clones in which *htrA* (1-1428 bp) and/or *secA* (2370-2598 bp) have been sequenced. **(A)** Schematic presentation of a chromosomal region *rocE-ispDF* in *H. pylori* wild-type and *htrA*-mutated strains. The genes are not drawn to scale. Features most important for analysis of the mutated strains are depicted. **(B)** PCR analysis of genomic DNA isolated from *H. pylori* wild-type and mutant strains. H6-H7 and H26-H27 primer pairs (S4 Table) was used to amplify *htrA* (upper panel, 1428 bp in wt strain, no PCR product in ΔhtrA strain) or *htrA* flanking regions (lower panel, 2316 bp in wt strain, 1712bp in Δ*htrA* strain) in selected kanamycin resistant pUZ16 transformants. **(C)** Analysis of 661T>G *htrA* mutation in *H. pylori* *htrA*S221A mutant strain. *htrA* was amplified by H6-H7 primer pair (upper panel, 1428 bp) and digested by PdiI (lower panel, PdiI digestion products only in *htrA*S221A strains). pUZ17 was used as a control of *htrA* amplification and PdIi digestion. Full length gels/blots are presented in Supplementary Fig. S7.

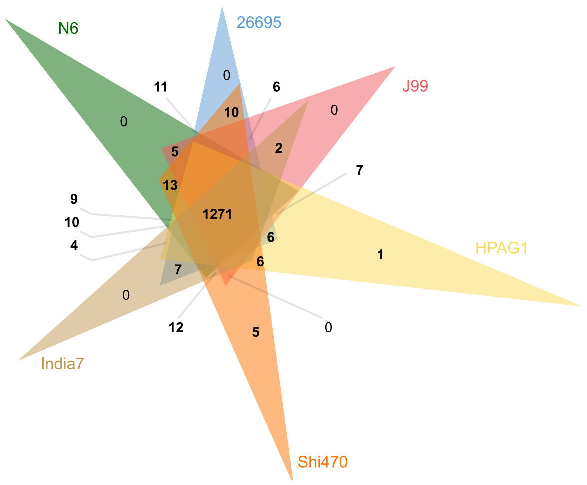

Fig S3. Comparison of orthologous genes among six *H. pylori* strains used in *htrA* mutagenesis. N6 proteome (defined by PROKKA 1.12) and UniProt proteomes of 26695 (UP000000429), J99 (UP000000804), HPAG1 (UP000008835), Shi470 (UP000008831) and India7 (UP000009059) were analysed using OrthoVenn. No orthologous gene cluster was found to be unique in *H. pylori* N6.

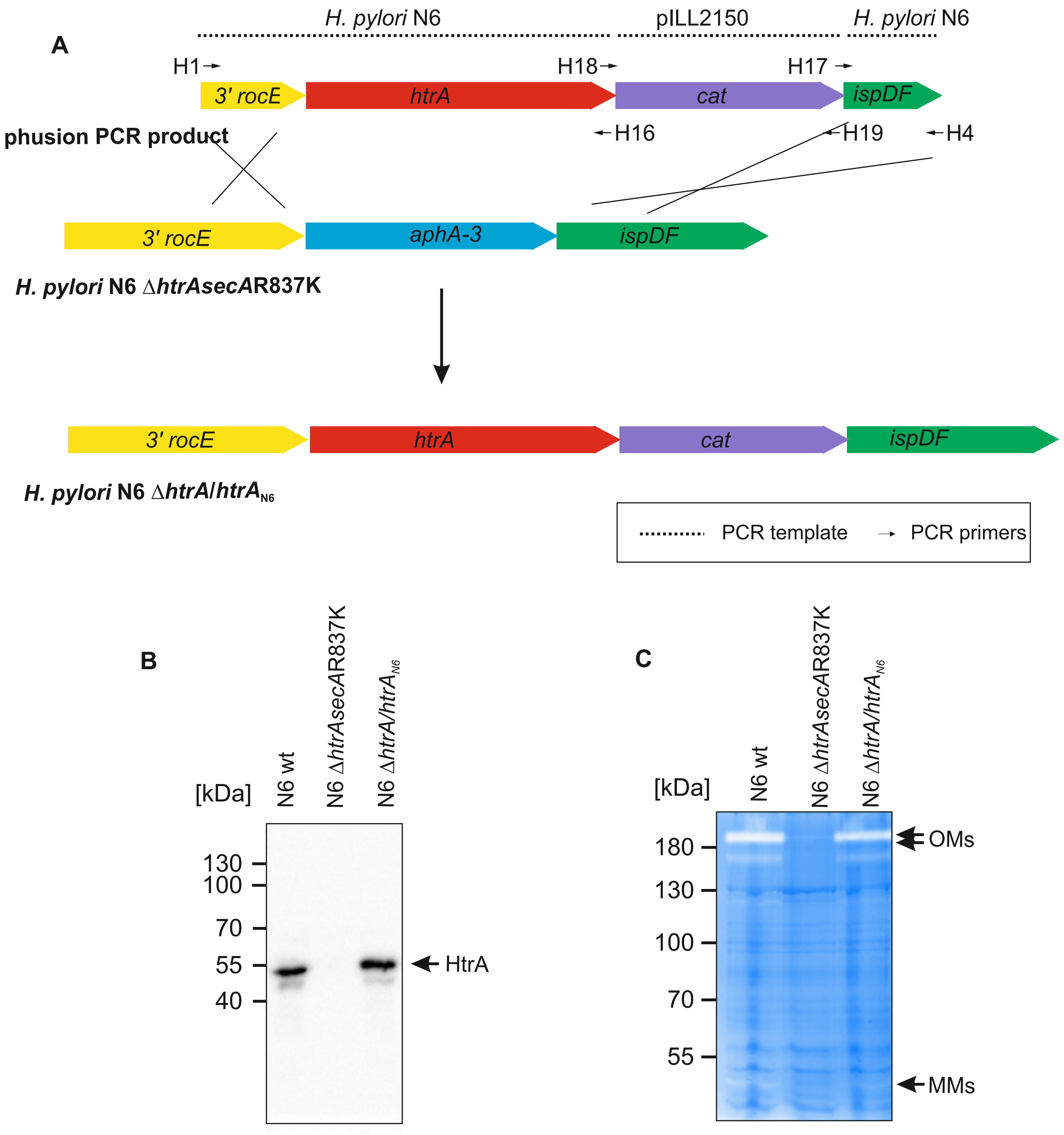

Fig. S4. Analysis of HtrA synthesis and activity in *H. pylori* Δ*htrA/htrA*$_{N6}$ complementation strain. **(A)** The mutagenesis strategy used to complement *htrA* on the *H. pylori* chromosome. The fusion PCR product recombined with the *H. pylori* N6 Δ*htrA* chromosome via double crossing over to give N6 Δ*htrA*/*htrA*$_{N6}$ complemented mutant strain. Primer sequences are given in S4 Table. **(B)** Western blot analysis of HtrA in *H. pylori* strains. A rabbit polyclonal anti-HtrA IgG was used to detect HtrA (51 kDa) in bacterial lysates.**(C)** The ability to cleavage of casein was analyzed by zymography. The position of proteolytically active HtrA monomers (MMs) and oligomers (OMs) is indicated. Full length gels/blots are presented in Supplementary Fig. S9.

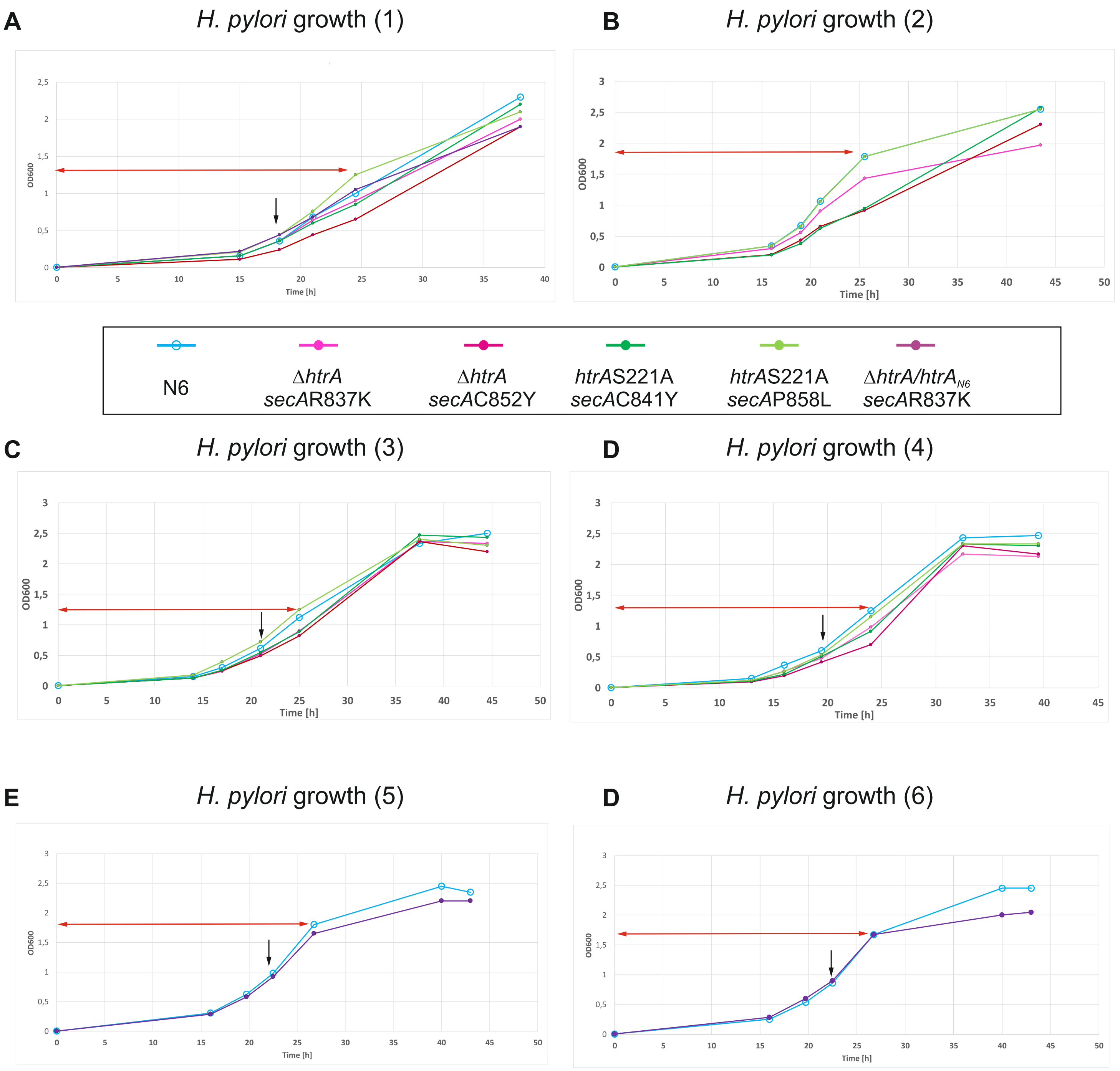

Fig S5. Growth curve analysis of *H. pylori* N6 wild-type and *htrA* mutant strains. *H. pylori* was inoculated in Brucella broth to OD_{600}=0.005 and cultured until stationary phase of growth (35-45 hours). Four independent analyses (A-D) were performed for *H. pylori* Δ*htrA* and *htrA*S221A mutant strains and three analyses (A, E, D) were done for Δ*htrA/htrA*$_{N6}$ complementation strain. Red arrows indicate time periods used in generation time calculations (Fig. 4B), while black arrows indicate time-points of samples collection for RNA isolation and *secA* expression analysis (Fig. 4C).

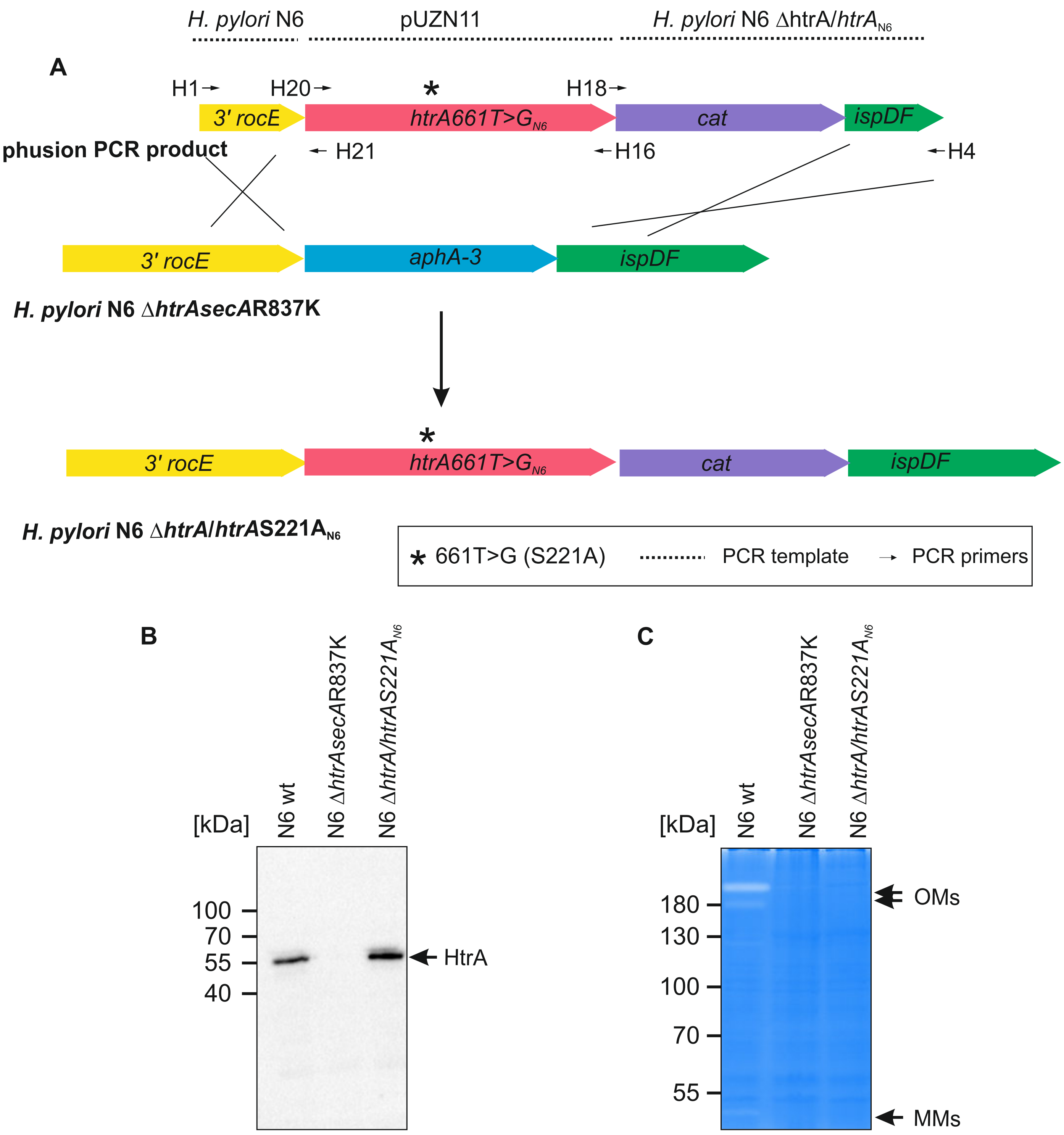

Fig. S6. Analysis of HtrA synthesis and activity in *H. pylori* Δ*htrA*/*htrA*S221A$_{N6}$ strain.
(A) The mutagenesis strategy used to introduce mutated *htrA* 661T>G into the *H. pylori* chromosome. The fusion PCR product recombined with the *H. pylori* N6 Δ*htrA* chromosome via double crossing over to give N6 Δ*htrA*/*htrA*S221A$_{N6}$ mutant strain. Primer sequences are given in S4 Table. **(B)** Western blot analysis of HtrA in *H. pylori* strains. A rabbit polyclonal anti-HtrA IgG was used to detect HtrA (51 kDa) in bacterial lysates. **(C)** The ability to cleavage of casein was analyzed by zymography. The position of proteolytically active HtrA monomers (MMs) and oligomers (OMs) is indicated. Full length gels/blots are presented in Supplementary Fig. S9.

S2B

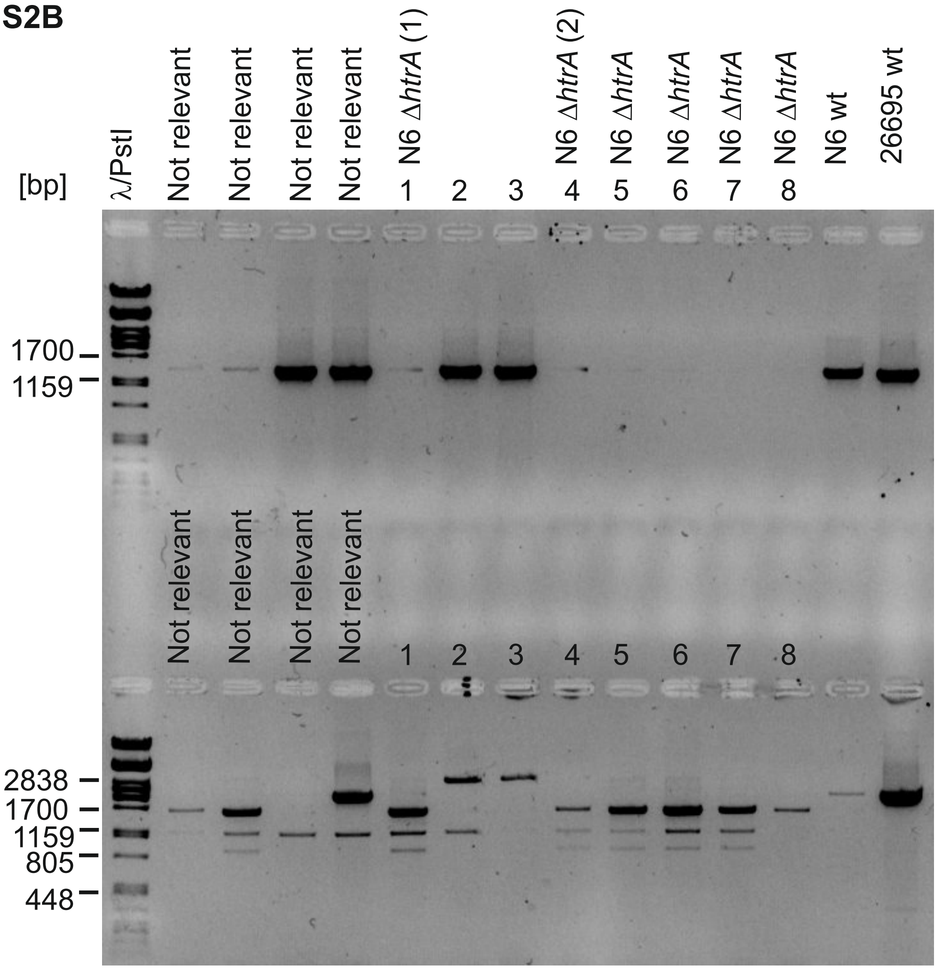

S2C

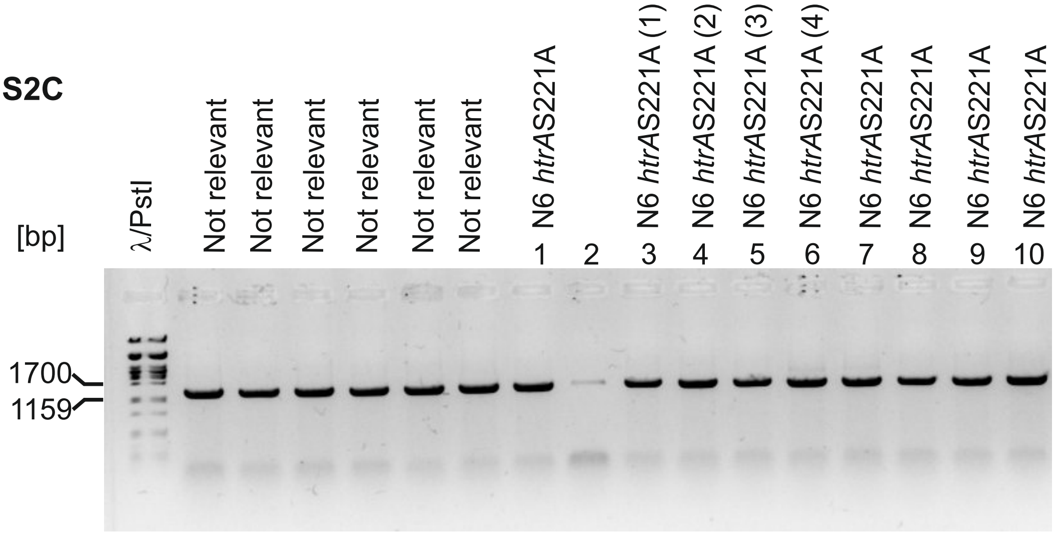

S2C

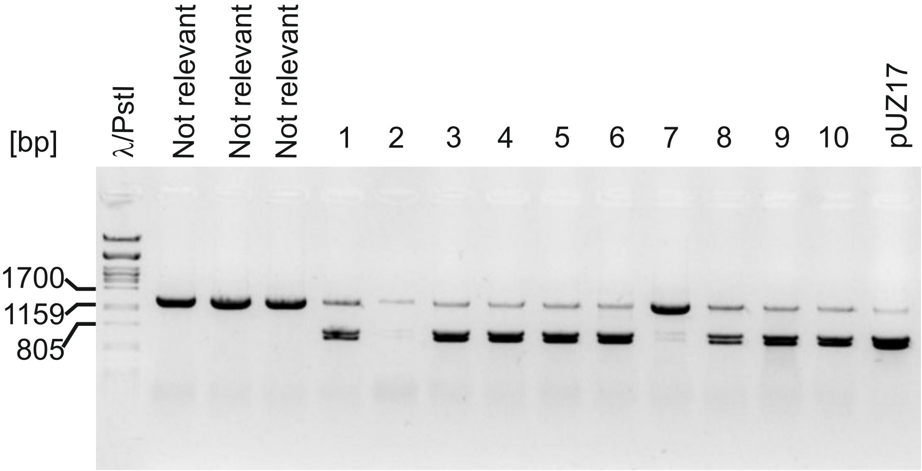

Fig. S7. Versions of the full length gels presented in Fig. S2. The number of the corresponding figure is presented on the left-hand side of each image; the lanes are described similarly as in Fig. S2; samples not relevant for this work are denoted „not relevant”.

Fig1B

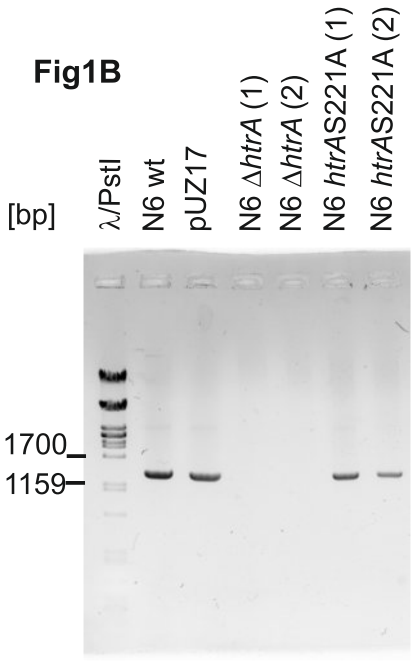

Fig1D

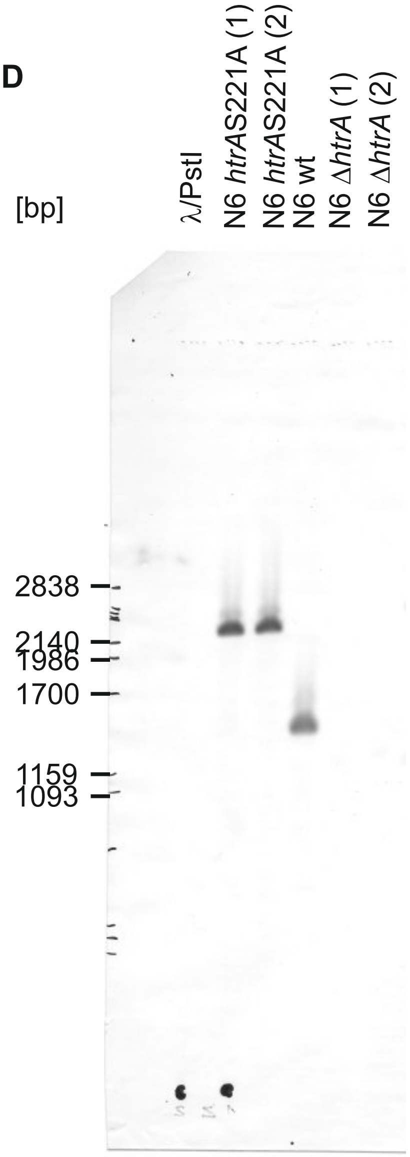

Fig2A

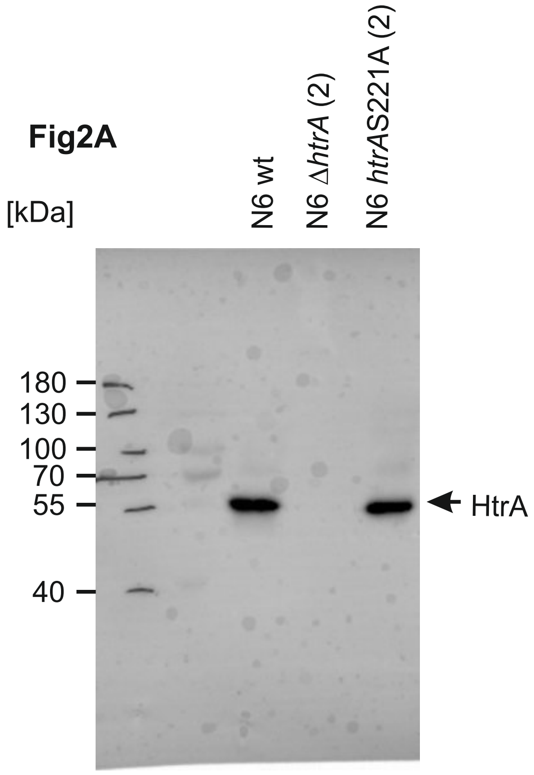

Fig2B

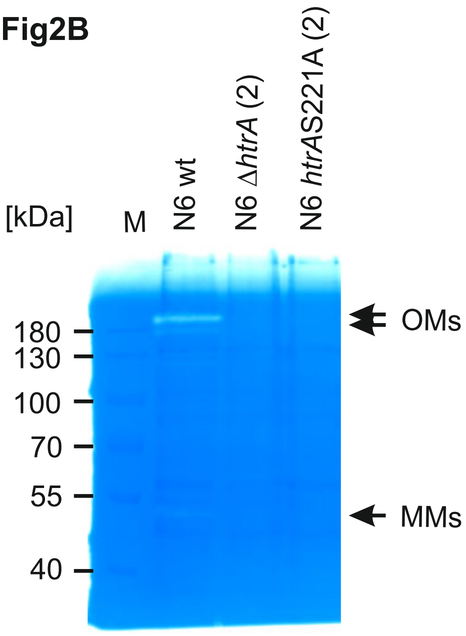

Fig. S8. Versions of full length gels/blots presented in Figs 1 and 2. The number of the corresponding figure is presented on the left-hand side of each image; the lanes are described similarly as in Figs 1 and 2. The images of a chemiluminescent blot and a marker were merged in section corresponding to Fig. 2A.

FigS4B

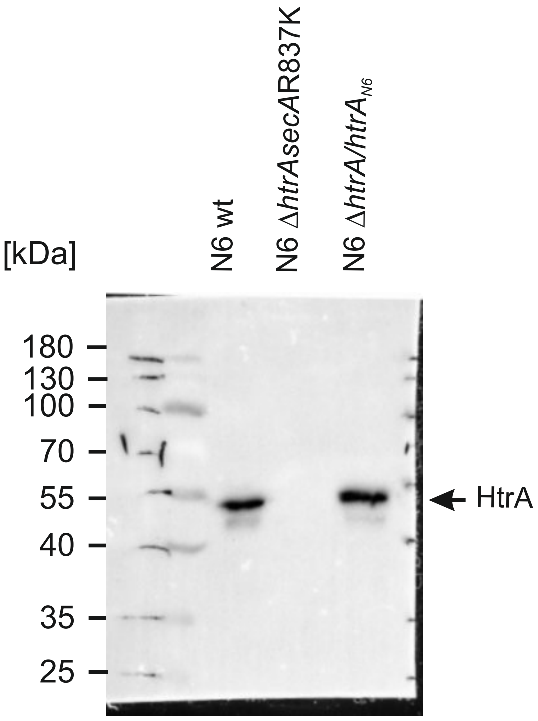

FigS4C

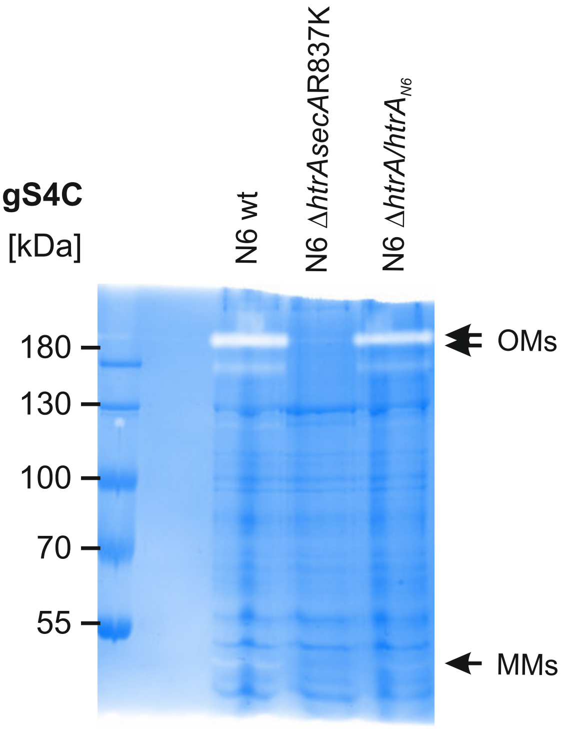

FigS6B

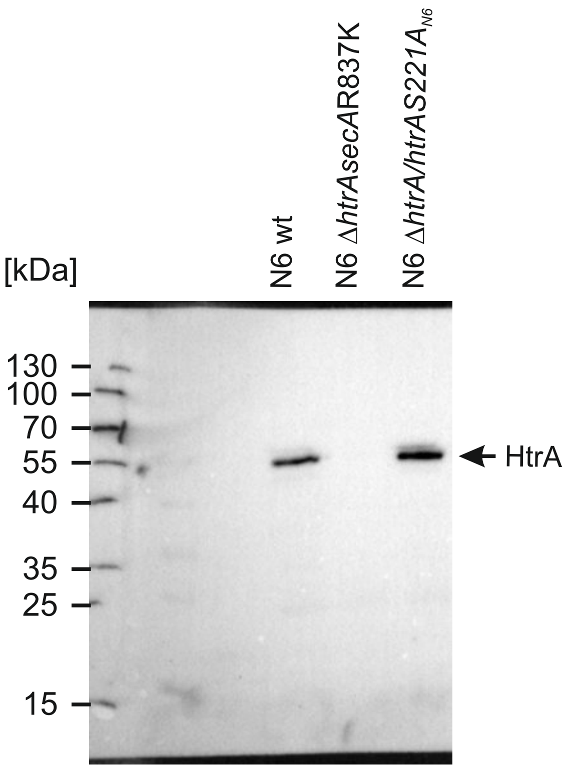

FigS6C

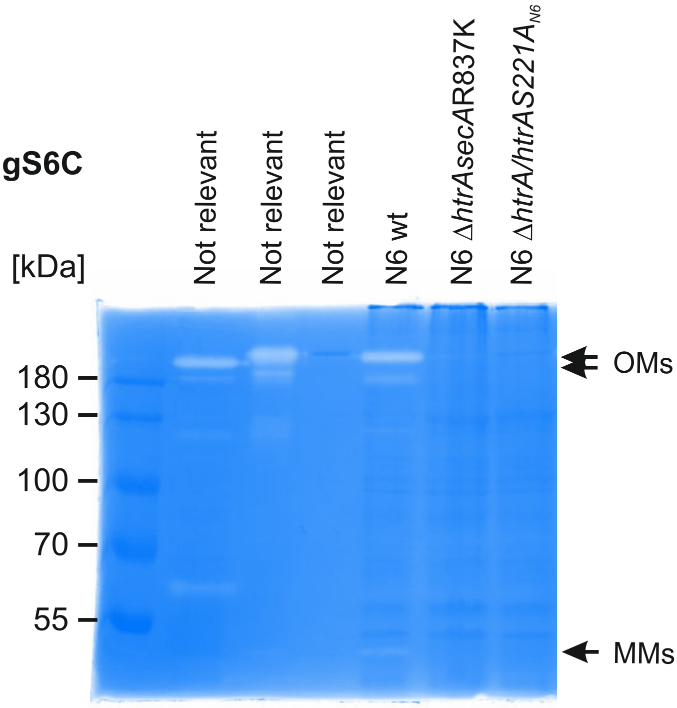

Fig. S9. Versions of full length gels/blots presented in Figs S4 and S6. The number of the corresponding figure is presented on the left-hand side of each image; the lanes are described similarly as in Figs S4 and S6; samples not relevant for this work are denoted „not relevant". Images of a chemiluminescent blot and a marker were merged in section corresponding to Fig. S4B and Fig. S6B.

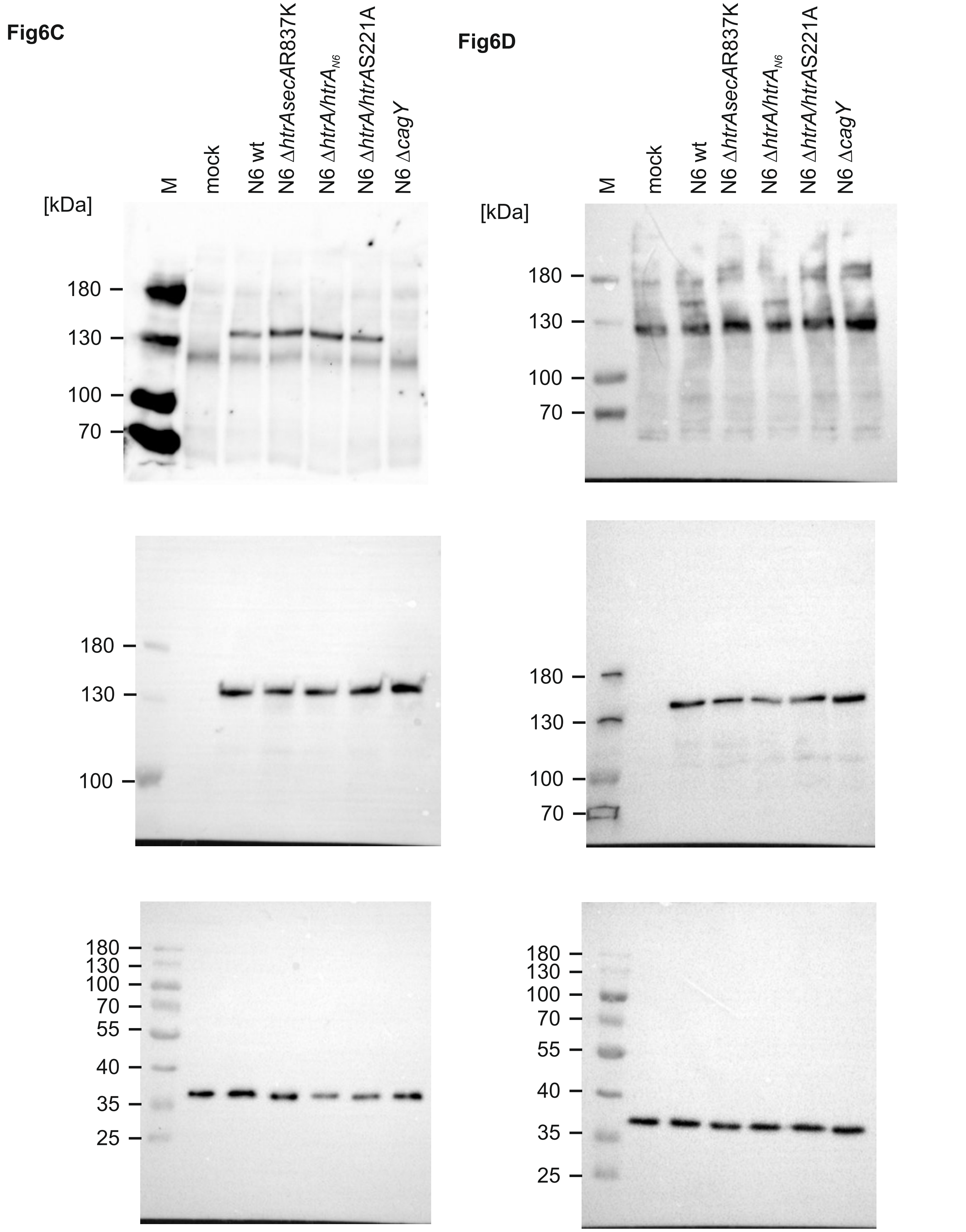

Fig. S10. Versions of full length blots presented in Fig 6. The number of the corresponding figure is presented on the left-hand side of each image; the lanes are described similarly as in Fig 6. Images of a chemiluminescent blot and a marker (M) were merged.
